# Loss of *Nod25* function does not affect nitrogen-fixing symbiosis in *Medicago truncatula*

**DOI:** 10.1007/s11103-026-01766-y

**Published:** 2026-10-08

**Authors:** János Barnabás Biró, Ágota Domonkos, Berivan Güngör, Attila Farkas, Elmira Mohammadi Eghbash, György Botond Kiss, Péter Kaló

**Affiliations:** 1 HUN-REN Biological Research Centre, Institute of Plant Biology, Szeged, Hungary; 2 Institute of Genetics and Biotechnology, Hungarian University of Agriculture and Life Sciences, Gödöllő, Hungary; 3 HCEMM-USZ Functional Cell Biology and Immunology Advanced Core Facility, University of Szeged, Szeged, Hungary; 4 AMBIS Biotechnol Res & Dev Ltd, Budapest, Hungary

**Keywords:** *Medicago truncatula*, Nitrogen-fixing symbiosis, Gene editing, Nodulin-25, IRLC legumes

## Abstract

The formation of nitrogen-fixing symbiotic nodules is a complex process that involves extensive transcriptional reprogramming of legume roots to enable intracellular accommodation of host compatible rhizobia. This process requires coordinated regulation of hundreds to thousands of genes, including the induction of highly expressed genes, traditionally referred to as nodulin genes. While the functions of many nodulins have been elucidated, a substantial number remained poorly characterized, or their functions are still unclear. One such gene, *NOD2*5, was originally identified based on differential hybridization between roots and nodules and is expressed at exceptionally high levels in Medicago nodules. MtNOD25-like proteins in IRLC legumes exhibit a repetitive modular structure flanked by conserved N- and C-terminal regions, with clade-specific repeat organization. To understand the role of NOD25 in symbiotic nitrogen fixation, we generated loss-of-function mutants in *Medicago truncatula* using CRISPR/Cas9-mediated genome editing. Four independent *Mtnod25* mutant lines displayed normal growth under symbiotic conditions and showed no detectable defects in nodule colonization, symbiosome formation, or bacteroid differentiation. These results indicate that *MtNOD25* is not essential for symbiosis in *M. truncatula* under the conditions tested. However, the biological function of MtNOD25 has not yet been elucidated, and its highly specialized evolutionary conservation suggests that it may have a specific, non-essential role in modulating or fine-tuning symbiotic processes under particular environmental or physiological conditions that remain to be defined.

**Supplementary Information:**

The online version contains supplementary material available at https://doi.org/10.1007/s11103-026-01766-y.

## Key message

Our results demonstrate that NOD25-like nodule-specific proteins possess conserved terminal domains and a rapidly evolving central region, suggesting functional diversification in IRLC legumes. Despite strong nodule-specific expression and symbiosome localization, loss of *MtNOD25* does not impair symbiotic nitrogen fixation, indicating that the protein is either non-essential under the conditions tested or functions in a context-dependent manner.

**Supplementary Information:**

The online version contains supplementary material available at https://doi.org/10.1007/s11103-026-01766-y.

## Introduction

Legumes have the ability to establish nitrogen-fixing endosymbiotic interactions with soil bacteria, known as rhizobia. During this process, symbiotic nodules develop on the roots of the host plant, where compatible rhizobia convert atmospheric nitrogen into a form that can be utilized by plants. This symbiosis enables legumes to grow and develop efficiently even in nitrogen-poor environments. Following mutual recognition and signal exchange between the partners, rhizobia attach to root hair cells and induce the formation of tubular structures, known as infection threads, through which they migrate into root cortical cells. These infection threads ramify and rhizobia are released and colonize the cytoplasm of nodule cells. Within these cells, the bacteria are enclosed by a plant-derived membrane called symbiosome membrane, where they multiply and differentiate into bacteroids. These endosymbiotic forms of rhizobia convert atmospheric nitrogen into ammonia (Zhang et al. 2024b). Legume root nodules have evolved into two major types, determinate and indeterminate, distinguished by the persistence of meristem activity (Nap and Bisseling 1990). Indeterminate nodules, typically formed on the roots of temperate legumes such as alfalfa, pea, clover and their close relatives retain an active meristem. As a result, these nodules develop an elongated, cylindrical shape. In contrast, determinate nodules, which are characteristic of many subtropical and tropical legumes, including *Lotus* spp., soybean and common bean, lose meristem activity after a defined period of time. Their subsequent growth occurs primarily with cell enlargement, leading to a spherical nodule structure.

Indeterminate nodules are characterized by a persistent apical meristem (Zone 1, Z1), which continuously generates new cells and establishes a spatial zonation corresponding to successive developmental stages of the symbiotic nodule (Vasse et al. 1990; Gavrin et al. 2014). In the infection zone (Zone II, ZII), rhizobia are released from infection threads into host cells, where they divide and become surrounded by plant cell-derived membrane, forming symbiosomes. In the transition zone (interzone, IZ), which consists of only a few cell layers, bacterial division ceases and both the bacteria and their host cells undergo differentiation. During this process, bacteria and host cells undergo multiple rounds of genome multiplication without cytokinesis. This results in elongated, genome-amplified bacteroids and enlarged, polyploid host cells that accumulate starch granules (Kondorosi et al. 2013). In the proximal and mature regions of the nodule, known as nitrogen fixation zone (Zone III, ZIII), differentiated bacteroids convert atmospheric nitrogen into ammonia. Nitrogen fixation takes place in a microaerobic environment that is established and maintained by leghemoglobin and a diffusion barrier located at the nodule periphery. Finally, at the base of older nodules, the senescence zone develops (Zone IV, ZIV), where infected cells and bacteroids undergo controlled degradation, enabling the remobilization of nutrients to the plant.

The interaction between the two partners induces a novel developmental program that involves rapid and massive transcriptomic changes (Pereira et al. 2024). These changes first occur in the legume root, initiating two developmental processes: the infection of root hair cells and nodule morphogenesis. Later, additional transcriptional changes take place in the developing and fully functioning nodules. The differential expression of symbiosis-related genes led to the concept of “nodulins” (Legocki and Verma 1980). The term “nodulin or nodule-specific genes” was used for genes whose activity could be detected in nodules but not in roots. Nodulin genes that are activated prior to the onset of nitrogen fixation are termed early nodulin genes, while those expressed mainly following the formation of nodule structure are called late nodulins, or simply nodulins (Van Kammen 1984).

Initially, nodulin genes were identified using differential hybridization of root and nodule cDNA libraries. Later, the comparison of complex gene expression profiles obtained from microarray analyses or total transcriptome sequencing (RNA-seq) enabled the systematic identification of nodule-specific genes (Gamas 1996; Fedorova et al. 2002; El Yahyaoui et al. 2004; Mergaert et al. 2020). Among the genes identified from a nodule-specific cDNA library based on their differential expression pattern, the alfalfa gene *Nms25* (*MsNOD25*) was one of the earliest discovered nodulin genes (Kiss et al. 1990; Végh et al. 1990). Subsequently, orthologs were identified in several other legumes, including Vicia species (*Nfv28/32* or *VfNOD28/32*; Küster et al. 1994, 1999), *Galega orientalis* (Kaijalainen et al. 2002), the model legume *Medicago truncatula* (Hohnjec et al. 2009) and red clover (Dinkins et al. 2022). In all examined cases, *NOD25* genes are strictly nodule-specific and highly expressed in nodules, accounting for ~ 1% of the total nodule transcriptome together with two other highly abundant transcripts, *leghemoglobin Lb120-1* and the *M. truncatula* ortholog of the *Nms32/34* nodulin gene (Burghardt et al. 2017). The product of *NOD25* orthologs are characterized by a repetitive modular structure flanked by conserved, unique N- and C-terminal regions, with the N-terminal sequence predicted to function as a signal peptide (Végh et al. 1990; Küster et al. 1996; Hohnjec et al. 2009). The central regions consist of few types of species-specific repetitive modules, each corresponding to a single exon. This genomic organization evolved by exon-shuffling (Patthy 1996) and frequently results in alternative splicing products lacking one or two repetitive modules (Küster et al. 1999). The *M. truncatula NOD25* gene (*MtNOD25*), which contains 13 exons, is exclusively expressed in infected nodule cells within the nitrogen-fixing zone. The functionality of the N-terminal signal peptide was demonstrated by the localization of an MtNOD25-GFP translational fusion to the symbiosome (Hohnjec et al. 2009). In addition, this was also shown by immunogold staining of MsNOD25 in *Medicago sativa* nodules (Brewin N and Kiss GB, unpublished results).

Despite extensive research, the biological function of NOD25 remains unresolved. Mutations in genes essential for nitrogen-fixing symbiosis can disrupt nodule development and function; therefore, mutant plants represent powerful tools for analyzing gene function in this process. However, no mutant alleles affecting NOD25 have been identified in the available insertion or deletion mutant collections of *M. truncatula*. Gene editing has recently emerged as a powerful approach for functional analysis of symbiotic genes in *M. truncatula* (Güngör et al. 2023; Zhang et al. 2024a; Saifi et al. 2024; Potsenkovskaia et al. 2024; Gao et al. 2025). Therefore, CRISPR/Cas9-mediated gene-editing was used to generate targeted mutations in *MtNOD25*.

In this study, we show that NOD25-like proteins containing central modular repeats are characteristic for cool-season legumes, and that several repetitive motifs are clade-specific. To investigate the function of MtNOD25, four mutant alleles were generated by targeted mutagenesis and regenerated into whole mutant plants; one represents a knock-out allele, while the other three are loss-of-function mutants. These mutant lines displayed no detectable symbiotic phenotype and showed no obvious differences in overall plant growth or morphology compared with wild-type plants. These results indicate that *MtNOD25* is dispensable for functional symbiosis under the conditions tested.

## Materials and methods

### Plant material, growth conditions, bacterial strains and nodulation assay

*Medicago truncatula* cv. Jemalong 2HA genotype (2HA) was used for transformation experiments. The mutant lines *dnf7-2*, *nad1-3*, and *Mtncr068* were used as control plants (Horváth et al. 2015; Domonkos et al. 2017; Güngör et al. 2023). Seeds were scarified and surface-sterilized by immersion in concentrated sulfuric acid for 8 min, followed by rinsing several times with distilled water. Sterilized seeds were vernalized for 3 days on inverted plates containing 0.8% (w/v) agar at 4 °C and subsequently germinated overnight at room temperature. For symbiotic assays, seedlings were grown in a zeolite substrate (Geoproduct Kft., Mád, Hungary) for 5 days prior to inoculation with rhizobia strains *Sinorhizobium medicae* WSM419 or *Sinorhizobium melilot*i FSM-MA. Bacterial cultures were grown overnight in liquid TA medium, harvested by centrifugation, and resuspended in nitrogen-free liquid Fahraeus medium (Fåhraeus 1957) to an OD_600_ of 0.1 prior to inoculation. Plants were maintained at 24 °C under a 16 h light / 8 h dark photoperiod.

### Design and assembly of CRISPR/Cas9 gene-editing constructs

Target sites for *MtNOD25* (MtrunA17Chr3g0100391) were identified using the CRISPOR online platform (Concordet and Haeussler 2018). Candidate 20-nt protospacer sequences beginning with G and followed by an NGG PAM were selected in the first two exons and ranked based on predicted off-target effects against the *M. truncatula* A17 reference genome (MtrunA17r5.0). The spacer sequence of the single-guide RNA (sgRNA) was cloned into the pKSE466-RR (Güngör et al. 2023) vector using annealed complementary oligonucleotides and assembled using Golden Gate cloning.

### Targeted genome editing and plant regeneration

Targeted genome editing and whole-plant regeneration were performed as previously described (Güngör et al. 2023). Briefly, the CRISPR construct pKSE466-RR-Nod25 was introduced into *Agrobacterium rhizogenes* ARqua1 for hairy root transformation. Transformed roots were selected based on DsRed fluorescence and maintained under sterile conditions prior to regeneration. For shoot regeneration, selected roots were transferred to callus-inducing medium (MTR-2) and incubated in the dark at room temperature for 2–3 weeks. Calli were subsequently transferred to MTR-3 medium supplemented with BAP and cultured at 24 °C under a 16 h light /8 h dark photoperiod. Emerging shoots were excised and transferred to MTR-3 medium to promote root development. Fully regenerated, rooted plants were transferred to zeolite substrate supplemented with Fahraeus medium containing 2 mM NH_4_NO_3_ for further growth.

### DNA isolation, PCR, and sequencing

Genomic DNA was isolated from the leaves of regenerated T1 and T2 plants using the ZenoGene40 Plant DNA Purification Kit (Zenon Bio, Szeged, Hungary) according to the manufacturer’s instructions. The genomic region encompassing the *MtNOD25* target site was amplified using 2× Phusion Master Mix (Thermo Fisher Scientific, Waltham, MA, USA) with the Nod25_F1 and Nod25_R1 primer pair. PCR products were subjected to Sanger sequencing. To determine the deletion boundaries in the *Mtnod25-2* mutant line, the Nod25_F10 and Nod25_R10 primers were used. The sequences of the amplicons from *Mtnod25-1*, *Mtnod25-3*, and *Mtnod25-4* lines were validated by Sanger sequencing.

### RNA extraction and RT–qPCR

For gene expression analysis, nodules induced by *S. medicae* WSM419 were harvested and ground in liquid nitrogen. Total RNA was extracted using TRI Reagent (Sigma-Aldrich), followed by purification with the Direct-zol RNA MiniPrep Kit (Zymo Research, Irvine, CA, USA), including on-column DNase I treatment according to the manufacturer’s instructions. RNA concentration was measured using a NanoDrop 1000 (NanoDrop Technologies, Wilmington, DE, USA), and RNA integrity was verified by agarose gel electrophoresis. First-strand cDNA was synthesized from 1 µg of total RNA using the SuperScript IV First-Strand Synthesis System (Thermo Fisher Scientific) with oligo(dT) primers. Quantitative real-time PCR (RT–qPCR) was performed on a LightCycler 96 (Roche, Basel, Switzerland) using qPCRBIO SyGreen Mix (PCR Biosystems, London, UK). Cycle threshold (Ct) values and relative expression levels were calculated using LightCycler 96 SW1.1 software. A ubiquitin-domain-containing gene (MtrunA17_Chr3g0126781) was used as the internal reference for normalization. Primers used for RT-qPCR are listed in Table S1.

### Confocal fluorescence microscopy

For fluorescence microscopy, nodules were harvested and immediately immersed in ice-cold 1× phosphate-buffered saline (PBS, pH 7.4), followed by fixation in 4% (w/v) paraformaldehyde. After fixation, samples were embedded in 5% (w/v) agarose blocks prepared in 1× PBS. Longitudinal sections (100 μm thick) were generated using a Leica VT1200S vibratome. Sections were incubated for 30 min at room temperature in 1× PBS containing 5 µM SYTO 13 (Thermo Fisher Scientific, Waltham, MA, USA). Fluorescence images were acquired using a VisiScope Spinning Disk Confocal Microscope with 488 nm excitation. Signal detection was performed using an Andor Zyla 4.2 PLUS.

### Scanning electron microscopy

Excised nodules with attached root fragments were rinsed in 1× PBS (pH 7.4), and their surfaces were gently scored with a hypodermic needle to facilitate fixative penetration. Samples were fixed in ice-cold solution containing 2.5% (v/v) glutaraldehyde in 1× PBS (pH 7.4) supplemented with 0.05 M cacodylate buffer, and incubated for 48 h at 4 °C. Following three washes in PBS, samples were embedded in 5% (w/v) agarose, and 100 μm longitudinal sections were prepared using a MICROM HM 650 V vibratome. Sections were dehydrated in a graded ethanol series (30–100%; 120 min per step), followed by a graded absolute ethanol– hexamethyldisilane (HMDS) series (3:1, 1:1, 1:3; 60 min per step), and a final 240 min incubation in pure HMDS. After air-drying in a fume hood, samples were mounted on SEM stubs using double-sided carbon tape and sputter-coated with ~ 12 nm gold using a Quorum Q150T. Imaging was performed with a Zeiss Sigma 300 FE-SEM equipped with a secondary electron (SE) detector, operated at 2 kV. Large-area mosaic images were acquired using the Atlas 5 system at a resolution of 50 nm per pixel.

### Phylogenetic and motif analysis

MtNOD25 homologous sequences from legume species were identified using BLAST searches based on conserved N- and C-terminal motif–encoding sequences. *De novo* gene annotation was performed using the FGENESH online tool (Softberry; https://softberry.com) (Solovyev et al. 2006). Conserved peptide motifs were identified using MEME Suite v5.5.8 (https://meme-suite.org/meme/index.html) (Frith et al. 2008; Bailey et al. 2015) with default parameters. For this analysis, the N-terminal conserved region, C-terminal conserved region, and internal regions were analyzed separately. For phylogenetic analysis, multiple sequence alignments of protein sequences were generated using Clustal Omega with default parameters and subsequently analyzed in MEGA v12 (Kumar et al. 2024). Phylogenetic relationships were inferred using the Maximum Likelihood method with the Jones-Taylor-Thornton model (Jones et al. 1992). Positions with less than 70% site coverage were excluded using the partial deletion option. Branch support values were estimated using the adaptive bootstrap method.

## Results

### The modular core structure of late nodulin NOD25 is restricted to IRLC legumes

**Fig. 1:**
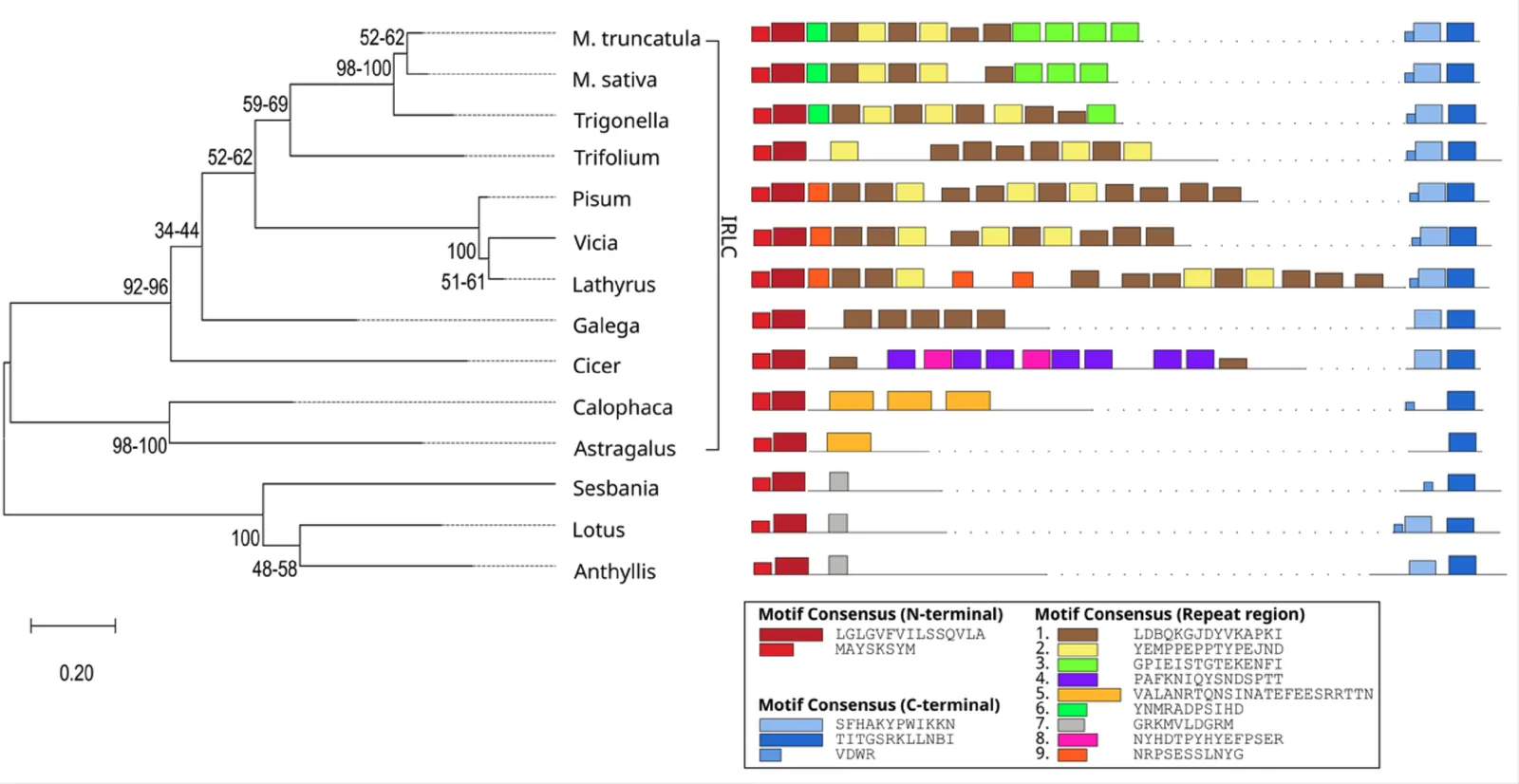
The phylogeny and structural organization of NOD25-like sequences in legumes. Phylogenetic tree of NOD25-like proteins identified in Hologalegina legume species (on the left). Full-length amino acid sequences of *Medicago truncatula* NOD25 homologs were aligned using the Clustal Omega program and the phylogenetic tree was generated using maximum likelihood evolutionary analysis. Branch support values indicate the percentage of replicates based on adaptive bootstrap analysis. Conserved motifs of NOD25-like peptide sequences from each species (*Medicago truncatula*, *Medicago sativa*, *Trigonella corniculata*, *Trifolium pratense*, *Pisum sativum*, *Vicia faba*, *Lathyrus tuberosus*, *Galega orientalis*, *Cicer arietinum*, *Calophaca sinica*, *Astragalus membranaceus*, *Sesbania bispinosa*, *Lotus japonicus*, *Anthyllis vulneraria*), identified using MEME Suite v5.5.8, are shown next to the phylogenetic tree. The N-terminal conserved regions (red), the C-terminal conserved regions (blue), and the internal regions (different colors) were analyzed separately. Brown boxes denote Repeat 2 modules, light yellow boxes indicate Repeat 1 modules, and light green boxes represent Repeat 3 modules, as previously described in *M. truncatula* (Hohnjec et al. 2009). Boxes are not drawn to scale

The *MtNOD25* gene encodes a protein characterized by unique N- and C-terminal sequences flanking a modular central core region (Hohnjec et al. 2009). Proteins with similar structural organization and composition are also present in the genomes of other legumes closely related to *M. truncatula* (Végh et al. 1990; Küster et al. 1999; Dinkins et al. 2022). To identify and study MtNOD25 orthologous proteins in other legume species, the highly conserved N-terminal sequences, which encode predicted signal peptides in all cases, and C-terminal amino acid sequences were used as queries to search legume databases. Homologous sequences were identified in legume species belonging exclusively to the Hologalegina clade (often referred to as “cool-season” or “temperate” legumes; (The Legume Phylogeny Working Group et al. 2013) (Fig. 1, Fig. S1a), and these sequences exhibited a high degree of similarity to the corresponding N- and C-termini of MtNOD25 (Fig. S2). In contrast, the analysis of the full-length protein sequences revealed that central core regions were less conserved and, depending on the phylogenetic distance from *M. truncatula*, contained either fewer or highly diverged repetitive sequences (Fig. 1, and Fig. S3). Altogether nine different repeat motifs were identified in the core region of NOD25-like proteins. Two of them, Motifs 1 and 2, occur almost exclusively in the Vicioid clade of legumes (The Legume Phylogeny Working Group et al. 2013). Motif 3 was specific to *Medicago* and *Trigonella* species, whereas Motif 9 was present only in the genera of *Lathyrus*, *Pisum* and *Vicia* in single or low copies in these genomes (Fig. 1 and S3). *Galega orientalis* lacked all motifs except Motif 1. In contrast, chickpea (*Cicer arietinum*) retained only a weak conserved version of Motif 1 but possessed entirely novel, genus-specific internal repeats. Similarly, *Calophaca sinica* and *Astragalus membranaceus* species lacked both Motif 1 and Motif 2, but contained lineage-specific repeat types (Fig. S3). Notably, in more distantly related species, such as *Lotus japonicus* or *Anthyllis vulneraria*, homologous sequences lacking any repetitive internal region were identified, suggesting that the presence of internal repeats is a distinctive feature of IRLC legumes.

Significant similarities are observed in the C-terminal regions of MtNOD25-like sequences across Hologalegina legumes, comprising highly and moderately conserved residues (Fig. S2), which delineate key motifs within this domain. In particular, two motifs, PWxKKN and GS/TRK/RLLL, separated by 4–6 residues, are strongly conserved and likely represent functionally important regions.

To further investigate the structural characteristics of NOD25-like proteins, we generated structure predictions using AlphaFold 3 (www.alphafoldserver.com; Abramson et al. 2024). The best-scoring models had pTM scores below 0.5, indicating low confidence in the predicted structures of all NOD25-like proteins (Fig. S1b) (Zhang and Skolnick 2004; Xu and Zhang 2010). Apart from the predicted signal peptide regions, the proteins could not be modelled with high confidence, suggesting that the remaining regions may contain substantial intrinsically disordered regions.

### *MtNOD25* is highly expressed in nodules

The alfalfa late nodulin *Nodulin-25* gene was previously shown to be active from 11 days post-inoculation (dpi) with *Sinorhizobium meliloti*, exhibiting transcriptional kinetics and expression levels comparable to those of the leghemoglobin genes (Kiss et al. 1990). To investigate the regulation of the *M. truncatula* ortholog of this gene (*MtNOD25*), we monitored *MtNOD25* expression kinetics using RT-qPCR and examined microarray data from the Medicago truncatula Gene Expression Atlas (Benedito et al. 2008). *MtNOD25* induction was first detected at 4 dpi with *S. medicae* WSM419 (SmWSM419) which increases strongly by 7 dpi and remained elevated at 10 and 14 dpi (Fig. S4a). Consistent with this pattern, microarray-based expression data showed that *MtNOD25* is active in nodules at 5 dpi with *S. meliloti* (Fig. S4b). The transcriptional activity of *MtNOD25* was further analyzed using RNA-sequencing data from distinct nodule zones obtained by laser-capture microdissection (Roux et al. 2014). *MtNOD25* transcripts were most abundant in the interzone and nitrogen fixation zone (Fig. S4c), consistent with previous in situ hybridization results (Hohnjec et al. 2009). Quantitative analysis of the reads also revealed that *MtNOD25* is highly expressed, with a transcript level significantly exceeding not only those of several selected *NCR* genes, but also the level of the *Leghemoglobin1* gene.

Taken together, these expression data suggest that MtNOD25 might function during the later stages of symbiotic nodule development and function.

### Generating mutations in *M. truncatula NOD25* using a CRISPR/Cas9-mediated genome editing system

**Fig. 2:**
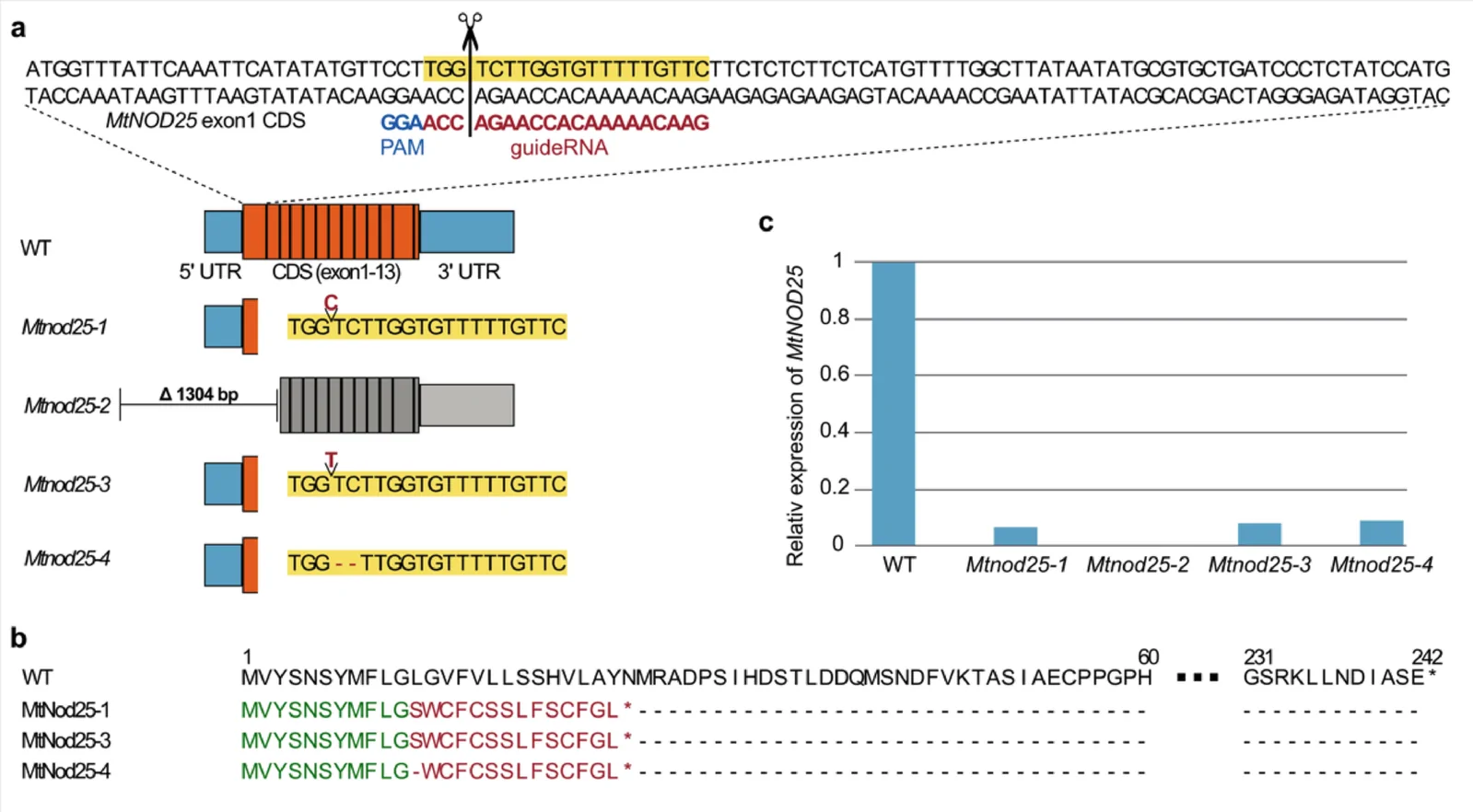
Targeted mutagenesis of *MtNOD25* with CRISPR/Cas9 gene editing. (**a** and **b**), The guideRNA target site was designed within the first exon of *MtNOD25*. The figure shows the nucleotide sequences of the obtained mutants, the corresponding deduced amino acid sequences, and the predicted structures of the gene products for the four *Mtnod25* mutant alleles. **c** Relative expression of *MtNOD25* in *Mtnod25* mutant nodules compared with wild-type nodules at 3 weeks post inoculation with *S. medicae* strain WSM419

To investigate the functional role of *MtNOD25* in nitrogen-fixing symbiosis, we employed CRISPR/Cas9 gene editing to generate mutant alleles of *MtNOD25*. A guide RNA (gRNA) targeting the first exon of *MtNOD25* was designed (Fig. 2a) and cloned into the single-guide RNA construct as previously described (Güngör et al. 2023). To induce Cas9 endonuclease-mediated mutations, the construct was introduced into roots of *M. truncatula* Jemalong 2HA genotype using *A. rhizogenes*-mediated hairy root transformation. Transformed roots, identified by the red fluorescent protein (DsRed), were collected 4–6 weeks post transformation for genotyping. The targeted region of *MtNOD25* was PCR-amplified and sequenced from root samples. Simultaneously, the hairy root segments were placed on regenerative medium. We successfully regenerated plants and obtained seeds from four independent lines carrying mutations in *MtNOD25*. Two lines, *Mtnod25-1* and *Mtnod25-3*, contained single base-pair insertions, while gene editing produced a 2-bp deletion in *Mtnod25-4* (Fig. 2a). These mutations introduced non-MtNOD25-specific residues after the mutation site and premature translational termination codons, resulting in truncated proteins lacking both the complete modular core region and the C-terminal domain of NOD25 (Fig. 2b). In an additional allele, *Mtnod25-2*, a 1304-bp deletion beginning 843 bp upstream of the predicted ATG start codon and ending 461 bp downstream of the translational start site in the second intron removed most part of the promoter and the first two exons of *MtNOD25* (Fig. 2a and S5).

To assess how the mutations affected *MtNOD25* activity, transcript levels were quantified in nodules 3 weeks post-inoculation (wpi) with SmWSM419 using RT-qPCR. A strong reduction in the relative transcript level of *MtNOD25* was detected in all four mutant alleles, with expression completely abolished in *Mtnod25-2*, the line with the promoter deletion (Fig. 2c). Based on gene expression data and predicted protein structures, *Mtnod25-2* was classified as a knockout mutant, whereas *Mtnod25-1*, *Mtnod25-3*, and *Mtnod25-4* were considered truncated mutants of *MtNOD25*.

### *Mtnod25* mutants exhibit wild-type-like nodule development

**Fig. 3:**
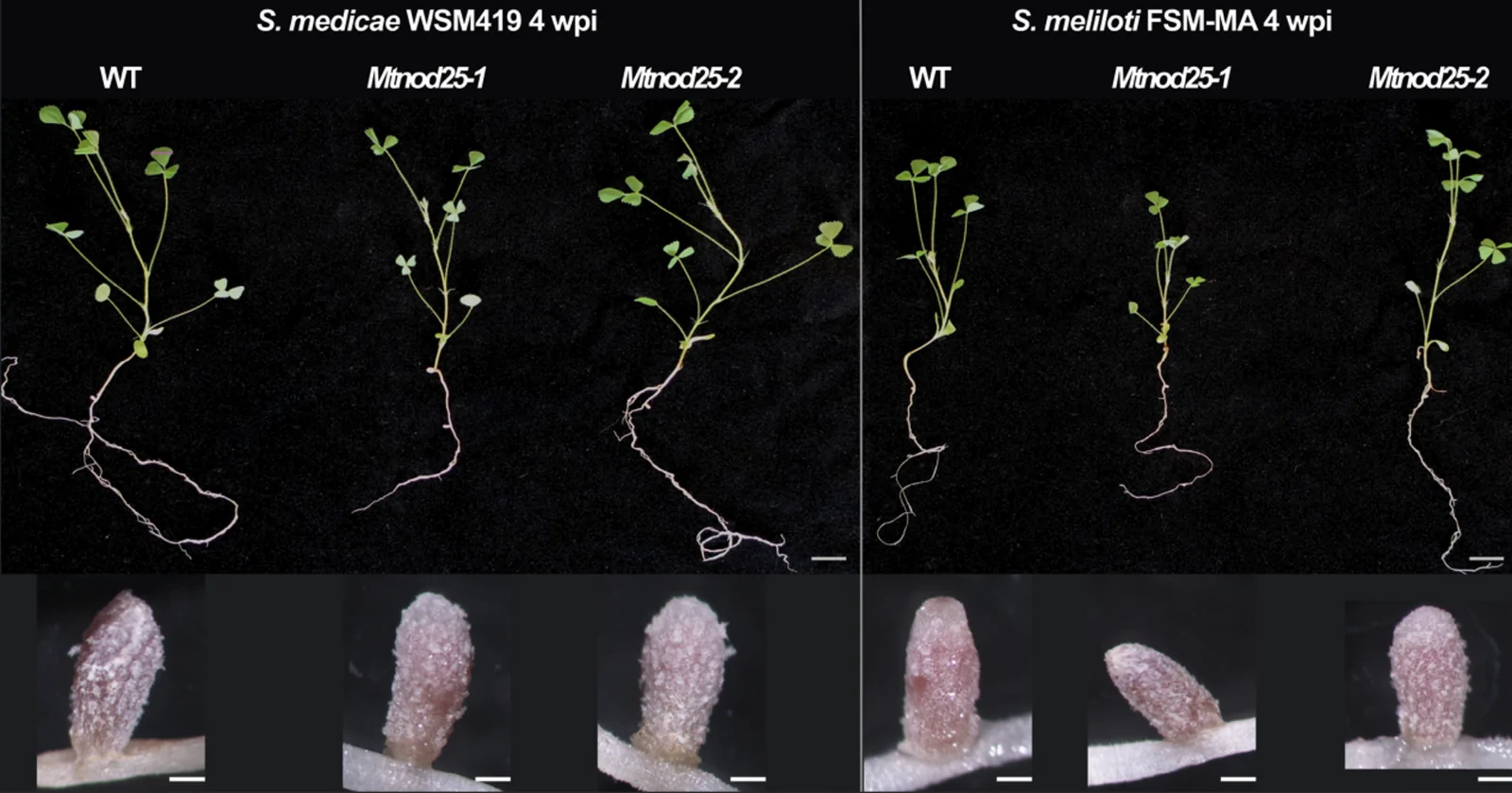
*Mtnod25* mutants show normal plant and nodule morphology compared to wild-type plants. 4 weeks post inoculation with rhizobia strains *S. meliloti* WSM419 and *S. meliloti* FSM-MA. Wild-type, *Mtnod25-1* and *Mtnod25-2* plants developed pink cylindrical nodules characteristic of functional nitrogen-fixing symbiosis. Scale bars: 2 cm (whole plants) and 0.5 mm (nodules)

*MtNOD25* is highly expressed in the infected nodule cells throughout the interzone and nitrogen-fixing zone (Fig. S4) (Hohnjec et al. 2009; Roux et al. 2014), suggesting that it may play a role in the late stages of the symbiotic interaction. To assess the symbiotic phenotype of *Mtnod25* alleles, mutant plants were inoculated with *Sinorhizobium meliloti* FSM-MA (SmFSM-MA) and *S. medicae* WSM419 (SmWSM419), and the gross phenotype of mutant plants and nodule morphology were analyzed 4 wpi. Under symbiotic conditions, both wild-type and *Mtnod25* mutant plants exhibited similar growth habits (Fig. 3 and S6) and developed dark green leaves without any visible signs of nitrogen starvation. Elongated pink nodules formed on mutant roots, resembling wild-type nodules in both size and morphology.

**Fig. 4:**
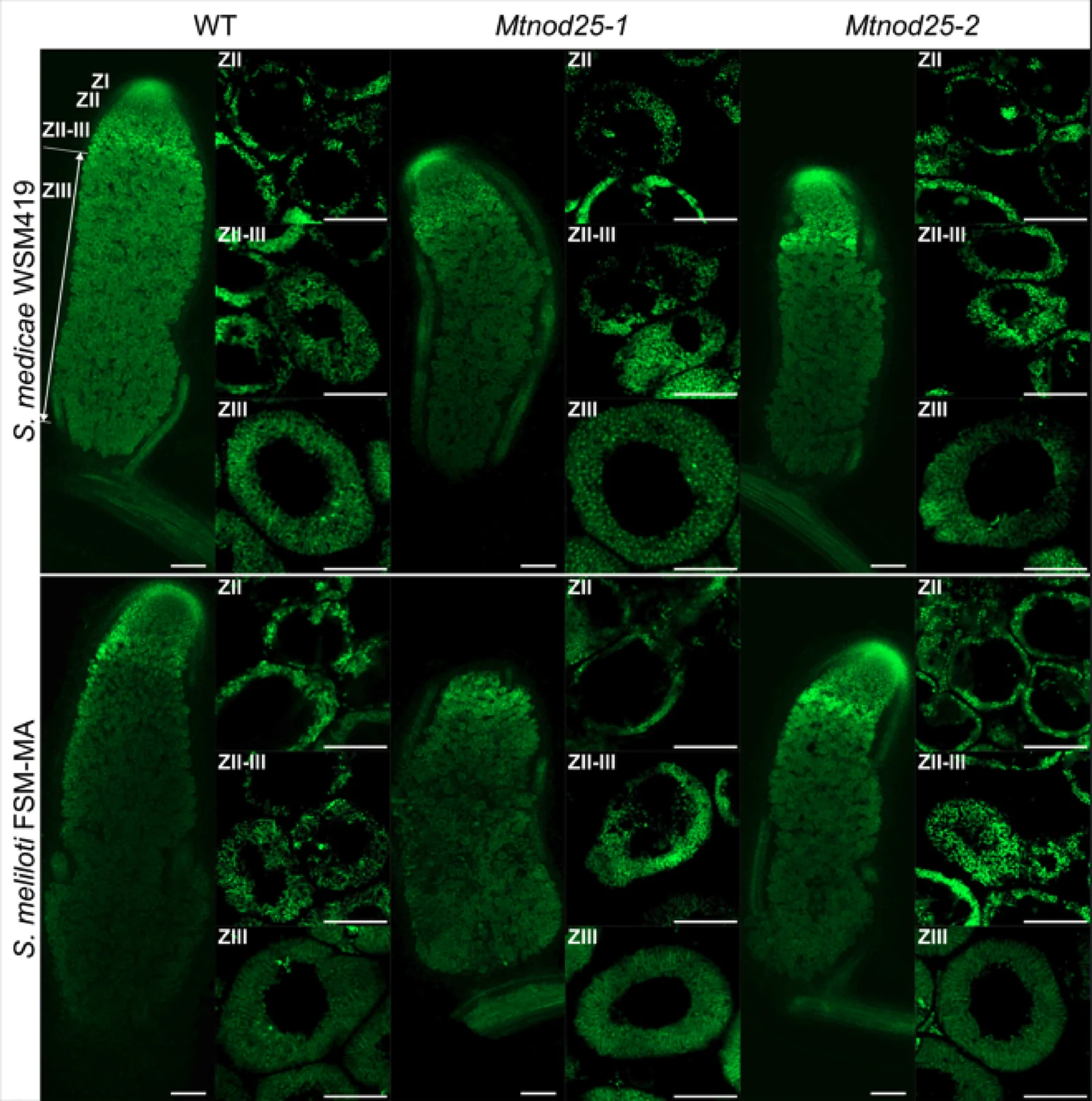
Rhizobia colonization and bacterial morphology in *M. truncatula* wild-type and *Mtnod25* mutant nodules. Colonization of symbiotic cells and bacteroid morphology were analyzed in SYTO13-stained longitudinal sections of wild-type, *Mtnod25-1* and *Mtnod25-2* nodule sections by spinning disc confocal microscopy 4 weeks post inoculation with *S. medicae* WSM419 and *S. meliloti* FSM-MA. *Mtnod25-1* and *Mtnod25-2* nodules were indistinguishable from wild-type nodules and exhibited the characteristic zonation of indeterminate nodules with colonized symbiotic cells. Higher magnification images show symbiotic cells from the infection zone (ZII), interzone (ZII-III) and nitrogen fixation zone (ZIII), revealing comparable stages of symbiotic cell differentiation and bacteroid morphology between wild-type and *Mtnod25* mutant nodules. Scale bars: 200 μm (nodules) and 20 μm (insets)

To investigate the colonization of symbiotic nodules, nodule sections from mutant and wild-type plants were stained with fluorescent nucleic acid-binding dye SYTO13 at 4 wpi with both rhizobia strains and examined using spinning disc confocal microscopy. Nodules of wild-type plants displayed the characteristic zonation of mature indeterminate nodules, with cells densely occupied by bacteria in zones II, II-III and III (Fig. 4). The organization of nodule zones, differentiation of symbiotic nodule cells, including vacuole enlargement in zone II, contraction in interzone and expansion in zone III (Gavrin et al. 2014), were essentially the same in *Mtnod25* nodules. Higher magnification revealed no notable differences in positioning or morphology of rhizobia (Fig. 4).

**Fig. 5:**
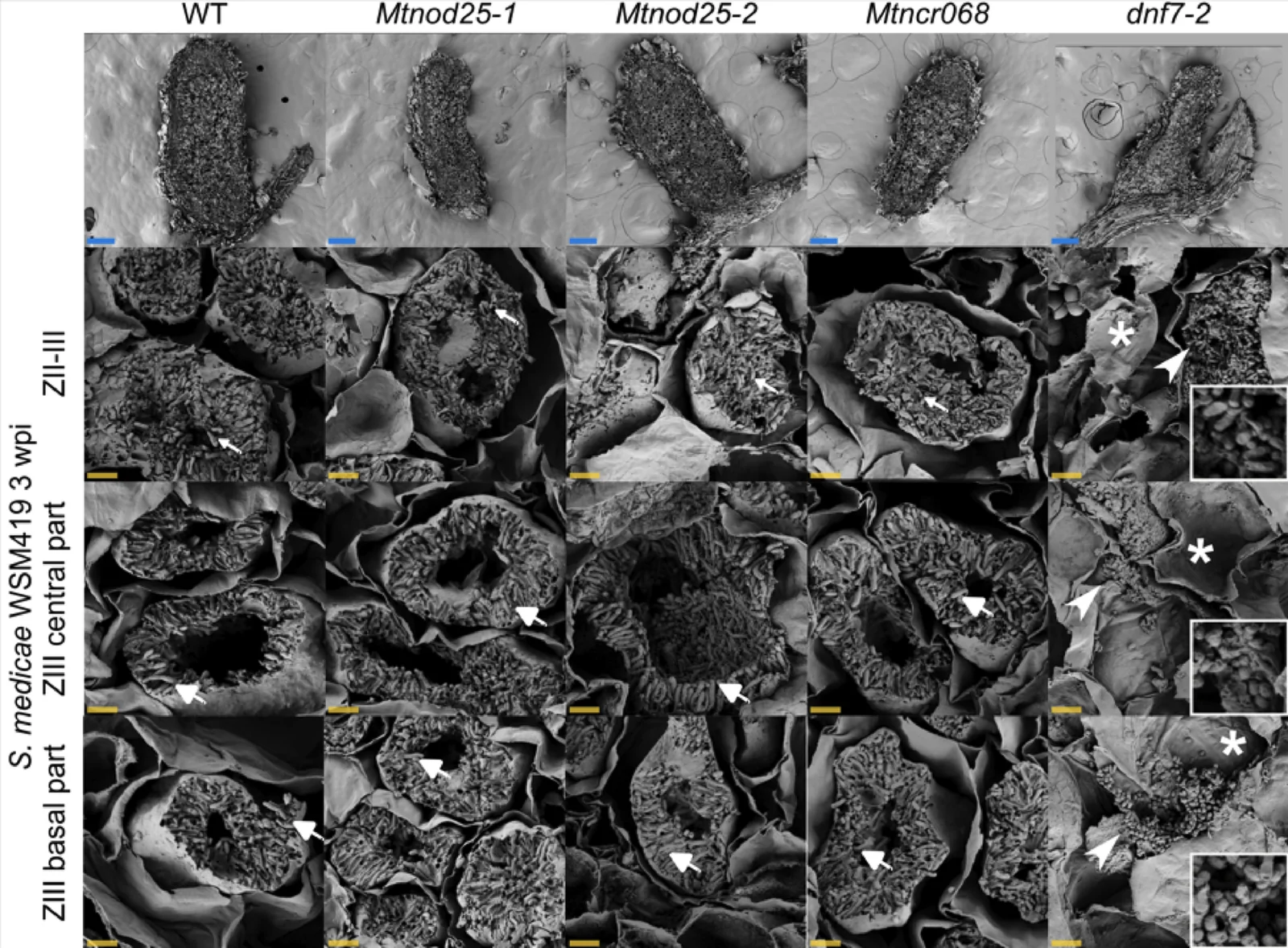
Symbiotic cells of *Mtnod25* nodules are indistinguishable from wild-type nodule cells. Scanning electron micrographs of nodules from wild-type, *Mtnod25-1*, *Mtnod25-2*, *Mtncr068* and *dnf7-2* plants collected 3 weeks post inoculation with *S. medicae* strain WSM419. Bacterial morphology was examined in high- magnification images of cells from the interzone (ZII-III) and the central or basal regions of nitrogen fixation zones (ZIII). Undifferentiated, round-shaped rhizobia (arrowheads) were observed in ZII-III and ZIII of the symbiosis defective *dnf7-2* nodules, whereas differentiated bacteroids (small and large arrows), positioned around the central vacuoles, were present in nodules of wild-type, *Mtncr068* and *Mtnod25* mutant plants. Asterisks show empty symbiotic cells in *dnf7-2*. Blue scale bars: 200 μm, yellow bars: 5 μm

To detect potential subtle alterations in *Mtnod25* mutant nodules, the ultrastructure of nodules of wild-type and mutant plants defective in nodule-specific genes was analyzed by scanning electron microscopy (SEM) 3 wpi with strains SmFSM-MA and SmWSM419. Two *Mtnod25* alleles were analyzed alongside the *Mtncr068* mutant, which establishes an effective nitrogen-fixing symbiosis despite disruption of the NCR068 peptide gene (Güngör et al. 2023), and *dnf7-2*, in which the loss of peptide NCR169 results in ineffective symbiosis with rhizobia (Horváth et al. 2015). Consistent with previous findings, *dnf7-2* mutant nodules contained infected cells with undifferentiated rod-shaped bacteria restricted to the infection and interzones (Fig. 5). Cells in the zone corresponding to the nitrogen fixation zone of wild-type nodules were devoid of rhizobia, instead, rod-shaped saprophytic bacteria released from infection threads were observed between cells or entangled with cell debris (Fig. 5 and S7) (Horváth et al. 2015). In contrast, the cells in the nitrogen-fixing zone of wild-type, *Mtnod25* and *Mtncr068* nodules were densely packed with elongated bacteroids oriented toward the central vacuoles. In agreement with light and fluorescent microscopy images, no visible differences were detected in the morphology and invasion of nodule cells between wild-type, *Mtncr068* and *Mtnod25* mutant nodules. These results suggest that defects in *MtNOD25* do not compromise nodule development or bacterial transition in *M. truncatula* nodules.

### The symbiotic competence is maintained in *Mtnod25* mutant plants

**Fig. 6:**
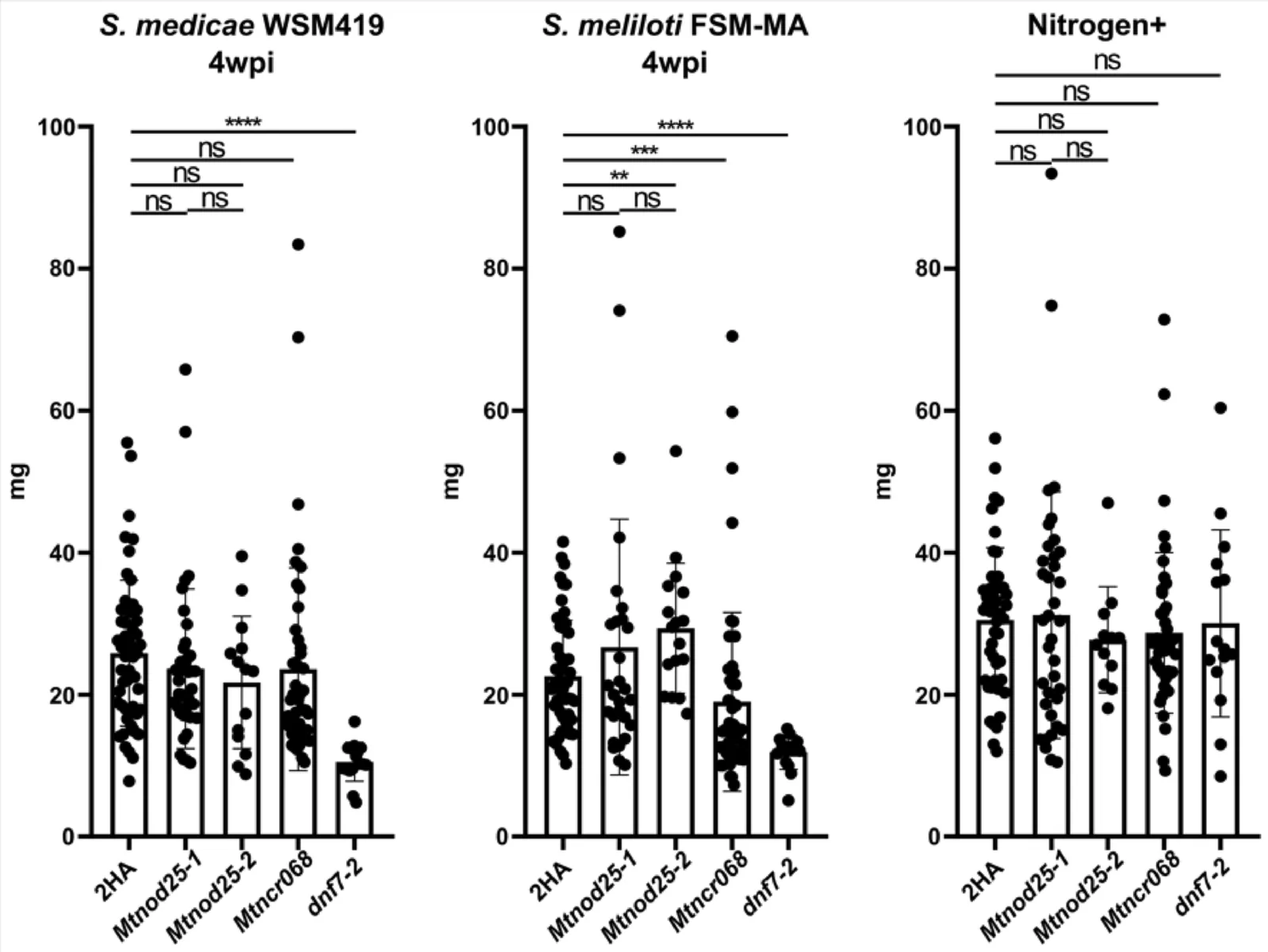
Shoot dry weight of *M. truncatula* wild-type and mutant plants deficient in nodule-specific genes. Dry weights of the aerial parts of wild-type and *Mnod25-1*, *Mtnod25-2*, *Mtncr068* and *dnf7-2* plants were measured 4 weeks post inoculation with either *S. medicae* strain WSM419 or *S. meliloti* strain FSM-MA or when grown in the same substrate without rhizobia and supplemented with 2.5 mM NH_4_NO_3_ (Nitrogen+). Bars represent ± SE of three biological replicates. Statistical significance is denoted as: ** *p* ≤ 0.01, *** *p* ≤ 0.001. **** *p* ≤  0.0001,

We did not observe any noticeable alterations in nodule development and colonization in *Mtnod25* mutants compared to wild-type plants. Therefore, we examined whether defects in *MtNOD25* cause any change in the symbiotic performance of the mutant plants. Two *MtNOD25* mutant alleles, along with two mutant lines defective in the NCR068 and NCR169 peptide genes, as well as wild-type plants, were inoculated with *S. meliloti* FSM-MA and *S. medicae* WSM419. In addition, the growth of these plants was analyzed under non-symbiotic conditions. The dry weight of the aerial parts of the plants was measured as an indicator of symbiotic effectiveness 4 wpi with rhizobia, or at the same time point for uninoculated but nitrogen-supplemented plants. The dry matter content of *Mtnod25*, *Mtncr068* mutant and wild-type plants inoculated with SmWSM419 was very similar, whereas the dry weight of *dnf7-2* plants was approximately 50% lower than that of the other lines (Fig. 6). When plants were inoculated with SmFSM-MA, a slight variation in dry weight was observed among wild-type, *Mtnod25* and *Mtncr068* lines; notably, the performance of the *Mtnod25* mutant plants slightly exceeded that of the wild-type. Under non-symbiotic conditions, with supplementation of 2.5 mM NH_4_NO_3_ and no rhizobia inoculation, the dry weights were essentially identical across all plant lines, indicating intact nitrogen assimilation in all mutant plants. These results demonstrate that the disruption of the *MtNOD25* gene does not affect the symbiotic performance of *M. truncatula* plants with either of the two tested rhizobium strains.

### *Mtnod25* nodules do not exhibit premature induction of senescence or pathogenesis marker genes

**Fig. 7:**
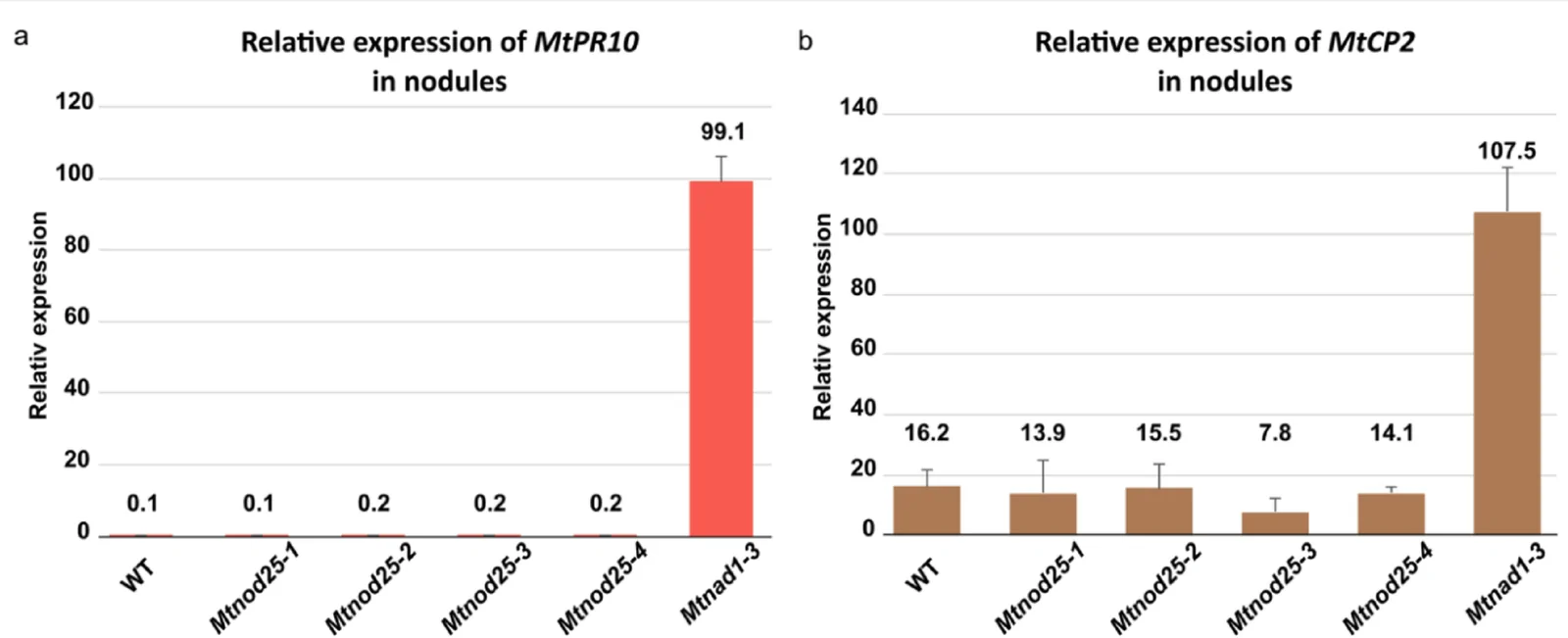
Defense-response and senescence marker genes are not induced in *Mtnod25* mutant plants. Relative abundance of the *MtPR10* defense-response marker gene (**a**) and the *MtCP2* senescence marker (**b**) in nodules of wild-type, *Mtnad1-3* and *Mtnod25* mutant nodules 3 weeks post inoculation with *S. medicae* strain WSM419. Values of relative transcript levels are the mean of three biological replicates. Error bars indicate ± SE

Although no signs of induction of early senescence or pathogenesis-related responses were observed on *Mtnod25* nodules 3 or 4 wpi with SmWSM419 or SmFSM-MA strains on SEM, light or spinning disc confocal microscopy images (Figs. 3, 4 and 5, S6 and S7), we wanted to make sure that genes involved in these processes were not activated. We monitored the activity of *MtPR10* (Class-10 *PATHOGENESIS-RELATED PROTEIN*), a pathogenesis-related marker gene and the *MtCP2*, a senescence response-specific cysteine protease gene (Pérez Guerra et al. 2010) in wild-type, *Mtnod25* and *Mtnad1-3* mutant nodules at 3 wpi using RT-qPCR. *Mtnad1-3* (*nodules with activated defense1–3*) is defective in controlling defence-like and senescence responses in symbiotic nodules (Domonkos et al. 2017) and a strong induction of both *MtPR10* and *MtCP2* was detected in *Mtnad1-3* mutant (Horváth et al. 2023). In contrast, we were unable to detect activation of either gene in *Mtnod25* mutants, similar to wild-type nodules (Fig. 7), indicating that neither early senescence nor defense response-specific pathways were activated in *Mtnod25* nodules.

## Discussion

The *NOD25* gene was originally identified by differential hybridization screening of alfalfa root and nodule cDNA libraries (Kiss et al. 1990) and several orthologs were subsequently isolated from closely related legume species (Küster et al. 1994, 1999; Hohnjec et al. 2009; Dinkins et al. 2022). In *Medicago* species, the expression level of *NOD25* genes is comparable to that of leghemoglobin genes, which represent some of the most abundant transcripts in the functional nodules (Kiss et al. 1990; Burghardt et al. 2017). Kinetic analysis revealed that *MtNOD25* is strongly induced at 7 days post-inoculation (dpi) with rhizobia and remains upregulated during the function of the symbiotic nodule. NOD25 proteins are targeted to symbiosomes, to the compartments wherein symbiotic nitrogen fixation occurs (Hohnjec et al. 2009) and proteomic studies identified MtNOD25 as a component of the symbiosome membrane fraction (Catalano et al. 2004). Together, these data suggest that NOD25 proteins may play a role in the functioning of the symbiotic nodule.

NOD25 proteins display a dual structural organization, consisting of conserved N- and C-terminal regions and a more variable central region. The N-terminal signal peptide of NOD25 shows strong similarity to the signal peptide of MtN22 and calmodulin-binding proteins MtCaML1-6, whose genes are clustered together in the *M. truncatula* genome (Liu et al. 2006; Hohnjec et al. 2009). Apart from these nodulins, no additional proteins with NOD25-like signal peptide have been identified in the *M. truncatula* genome by similarity searches, suggesting that this feature is specific to this nodulin gene cluster. Based on this observation, we searched for homologous proteins in other legume species using the conserved N- and C-terminal sequences to identify additional orthologs and characterize their structural features. NOD25-like sequences were found exclusively in Hologalegina legumes. Structural analyses of these proteins reveal a striking evolutionary duality, characterized by conserved terminal domains flanking a diversified central core. These results demonstrate that while the N-terminal signal peptide and the C-terminal sequence are conserved across the Hologalegina clade, the modular repetitive architecture is restricted to the Inverted Repeat-Lacking Clade (IRLC). This pattern suggests that the fundamental function of NOD25-like proteins was originally - and likely remains - determined by the terminal domains of ancient origin, while the central core has undergone substantial lineage-specific functional innovation. The absence of internal repeats in more distantly related species, such as *Sesbania bispinosa*, *Lotus japonicus* and *Anthyllis vulneraria*, suggests that the ancestral state of the NOD25-like protein was likely a simpler, non-repetitive form. The internal repeats likely arose by exon-shuffling after the divergence of the IRLC clade. The emergence of the modular core in the IRLC suggests a correlation with the unique symbiotic characteristics of this clade. It is noteworthy that the complexity of repeat motifs increases in accordance with phylogenetic distance from the base of the IRLC. For instance, Motif 1 is widespread in all IRLC legumes, the presence of Motif 2 is characteristic for IRLC legumes following the divergence of *Galega* species. The restriction of Motif 3 to the *Medicago* and *Trigonella* genera, and the unique, genus-specific internal repeats found in *Cicer arietinum*, point toward an ongoing process of molecular diversification. Furthermore, the divergence of motifs within the Vicioid clade - specifically the presence of Motifs 1, 2, and 9 in varying combinations - highlights the dynamic nature of the NOD25 gene family.

Such rapid evolution of internal repeats is frequently associated with proteins mediating specialized host-microbe interactions, where structural plasticity enables fine-tuning of biological functions. Prominent examples include proteins involved in host-pathogen recognition, often mediated by a large repertoire of rapidly evolving receptors, many of which contain leucine-rich or tetratricopeptide repeats (Andrade et al. 2001; Björklund et al. 2006; Zambounis et al. 2012). These repeat units are typically encoded by single exons, and often diversify by exon-shuffling mechanisms, resulting in variation in repeat numbers (Patthy 1996). In addition to the hydrophobic N-terminal signal-peptide, both the central and C-terminal regions of NOD25 are hydrophilic (Kiss et al. 1990; Küster et al. 1994), suggesting that the expansion of internal repeats in NOD25-like proteins may promote the formation of flexible secondary structures that enhance interaction surfaces or protein stability.

Given that IRLC legumes are characterized by the terminal differentiation of bacteroids, the restriction of the modular NOD25 structure to this clade, together with the presence of the rapidly evolving family of secreted NCR peptides in IRLC legumes (Montiel et al. 2017) and the strong nodule-specific expression of *NOD25*, raises the possibility that these proteins contribute to the specialized physiology of IRLC-type symbiosomes. To test this hypothesis and elucidate the function of NOD25, we generated gene-edited mutant alleles of *NOD25* in *M. truncatula*. Targeted mutagenesis of the first exon using *A. rhizogenes*-mediated hairy root transformation resulted in multiple independent loss-of-function alleles. The frameshift mutations observed in *Mtnod25-1*, *Mtnod25-3*, and *Mtnod25-4* introduce premature stop codons, resulting in truncated proteins lacking the significant portion of the conserved core region and the C-terminal domain, which are likely essential for protein function. The *Mtnod25-2* allele represents an even larger lesion, removing a substantial portion of the promoter and the first two exons, resulting in a null allele, as no transcript could be detected. The consistency of these independent loss-of-function alleles supports the robustness of the phenotypic analysis. Despite the strong expression of *MtNOD25* in infected nodule cells of wild-type plants, its loss did not impair nitrogen-fixing symbiosis in *M. truncatula*. *Mtnod25* mutant lines exhibited normal growth and symbiotic performance with the rhizobia strains tested. Mutant plants formed morphologically normal, fully colonized pink, leghemoglobin-containing nodules with typical zonation of indeterminate nodules. Bacteroid differentiation was indistinguishable from wild-type at both cellular and ultrastructural levels. No defects in bacterial transition or nitrogen fixation were observed, and molecular analyses did not reveal premature activation of senescence- or defense-related marker genes in mutant nodules. Together, these observations indicate that symbiotic nitrogen fixation remains fully functional in *Mtnod25* mutants. We therefore conclude that *NOD25* is not required for effective nitrogen-fixing symbiosis in *M. truncatula* under the conditions tested.

The absence of a detectable symbiotic phenotype in a mutant defective in a highly expressed nodulin is not unprecedented. Two decades ago, it was demonstrated that a natural mutant of diploid alfalfa plants lacking the *ENOD12* gene, strongly induced in nodules following rhizobia inoculation (Scheres et al. 1990), exhibited a wild-type symbiotic phenotype (Csanádi et al. 1994). *ENOD12* encodes a hydroxyproline-rich glycoprotein, and the *Medicago* genome contains additional genes encoding nodule-specific cell wall proline-rich proteins, such as ENOD11 (Journet et al. 2001). This redundancy suggests that other genes may compensate for the loss of *ENOD12* function. A similar situation has been reported for symbiotic leghemoglobin genes, which are encoded by a multigene family in legumes; only their combined silencing results in a detectable symbiotic phenotype proving the essential role of leghemoglobin genes for symbiotic nitrogen fixation in *L. japonicus* (Ott et al. 2005). A recent study applying CRISPR/Cas9 gene editing technology proved that the impairment of all three symbiotic leghemoglobin genes is required to obtain a leghemoglobin null mutant in *L. japonicus* (Wang et al. 2019). In contrast, sequence analyses have not identified homologues sequence for *MtNOD25*, suggesting a lack of functional redundancy. Therefore, it is less likely that other genes can compensate for its loss.

On the other hand, if NOD25 performs a similar biological function across the Hologalegina clade, this function is likely associated with the conserved C-terminal region. The N-terminal signal peptide is cleaved during protein targeting to the symbiosome, and species in the Robinioid clade (*Anthyllis*, *Lotus* and *Sesbania* sp.) possess only a single central domain, in contrast to the multiple repeat-containing IRLC clade. This suggests that the central region may primarily serve as a spacer, irrespective of its length and composition.

The sequence alignment revealed a conserved RK/RLLL motif in the C-terminal region of NOD25-like proteins. The RXXL or RXLX motifs are recognized as canonical cleavage sites for subtilisin-like serine proteases, also known as subtilases (Schaller et al. 2012). Subtilases are widespread serine peptidases in bacteria and eukaryotes and, beyond their role in general protein turnover, they are involved in the processing of peptides that regulate plant development, programmed cell death, and responses to biotic and abiotic stimuli.

Recent studies have highlighted the role of Brassicaceae-specific SCOOP peptides in regulating root elongation and defense responses (Gully et al. 2019; Hou et al. 2021). These peptides are derived from precursor proteins (PROSCOOPs), most of which contain an ‘RxLx/RxxL’ motif that is recognized and cleaved by specific subtilases (Yang et al. 2023), thereby resulting in bioactive signaling SCOOP peptides.

In legumes, the induction of subtilase (*Sbt*) genes has been reported in *L. japonicus* during both arbuscular mycorrhiza and rhizobia symbiosis (Kistner et al. 2005). One mycorrhiza-specific *Sbt* is active in root cells surrounding internal fungal hyphae, whereas another *Sbt* is continuously expressed during AM symbiosis but shows activity predominantly at early stages of rhizobia symbiosis. Furthermore, proteomic analysis of soybean nodules identified a subtilase protein in the peribacteroid membrane fraction, suggesting a potential role for subtilases in direct interaction with rhizobia (Panter et al. 2000). The conserved RK/RLLL motif in the C-terminal region of NOD25 proteins is consistent with a potential role in proteolytic processing. Such processing could provide a mechanism for NOD25 activation under specific environmental or physiological conditions and may explain the absence of a detectable symbiotic phenotype under standard conditions. One possibility is that NOD25 is present in the symbiosome as an inactive proprotein and is proteolytically processed only in response to particular biotic or abiotic stresses, such as pathogen infection, drought, flooding, salinity, or temperature stress. Under such conditions, the distinctive repeat architecture of NOD25 may confer a specialized function that is not required for symbiosis under standard conditions. Although this hypothesis could reconcile the evolutionary conservation of *MtNOD25* with the lack of an apparent symbiotic phenotype in the mutant lines, the proposed proteolytic processing and stress-dependent activation mechanism remain to be experimentally validated.

Our findings indicate that *MtNOD25* is dispensable for symbiosis under the conditions tested, they do not exclude a context-dependent or non-essential biological function. Notably, *NOD25*-like genes are highly expressed in nodules and retain intact protein-coding potential in legume genomes, with no evidence of nonsense mutation accumulation. Together with their evolutionary conservation, these features are consistent with selective constraint acting to maintain NOD25-like proteins. This conservation suggests that NOD25-like proteins may fulfil specialized functions under specific environmental or physiological conditions that remained to be identified.

## Supplementary Information

Below is the link to the electronic supplementary material.

Supplementary Material 1

## Data Availability

All data supporting the findings of this study are included within the manuscript and its supplementary files.
