# Supplementary material for: Loss of Nod25 function does not affect nitrogen-fixing symbiosis in Medicago truncatula
Source: Plant Mol Biol. 2026 Oct 8;116(5):101. doi: 10.1007/s11103-026-01766-y (PMC13649901; doi:10.1007/s11103-026-01766-y)
Supplement: Supplementary file 1 [file 11103_2026_1766_MOESM1_ESM.pdf]

a

```
CLUSTAL O(1.2.4) multiple sequence alignment
                                            SP
Sesbania              MAYSKS-YLCLGLGLFVILSSQALALQLPDAEATLPPGRKMT-----------LDGRME-    47
Lotus                 MASSKSYGLYLGLGLFVILFSQVFAHTLTEADKAADPERKMV-----------LDRRINM    49
Anthyllis             MGSSKSCDLYLGLGLLAILFSQVFSQTLPQNEVN-NLGEKMV-----------MDGRMSV    48
Calophaca             MAYSKSC-MPLVLGLFVILSSQVLAHKVPETLVPISDVALANHTQN-SINATEFEE----    54
Astragalus            MANLKTC-ISLGLGLLVILFSQVIADKSPEAICDSSLL--SCGSKN-SVEVAEFEE----    52
Vicia                 MAYSNSY-MLFLLGVSLIISSQVLAYNRPSKSSLSYGEALDEQKGLDYVNAPKMYEELEA    59
Pisum                 MAYSNSY-ILFLLGVSVILSSQVLAYNRPSESSLNYGEELDEQKGLDYVNAPKMNEELEA    59
Lathyrus              MAYSNSY-MLFLLGVSLILSSHVLAYNRPHESSLNYGEELDEQKGIDYVNAPKMNEELEA    59
Cicer                 MDNSKSY-MLLCIGLFVILCSQVLAYNDPVPPIC--DGELYYHKDLVYEKIPIIDGPPDV    57
Galega                MAYSKPH-MLLCLGFFVILSSNVLVDKVNCV-----------------------------    30
Trifolium             MVYSNSY-MFLGIGVFVILSSHALANHLASEPLI-YDVYKM-------------------    39
Trigonella            MVYSNSC-MFLGLGVFVLLSSHVLAYNMRADASI-YDSTLDDQKSINYVNVAST-----T    53
Medicago_truncatula   MVYSNSY-MFLGLGVFVLLSSHVLAYNMRADPSI-HDSTLDDQMSNDFVKTAS-------    51
Medicago_sativa       MVYSNSY-MFLGLGVFVLLSSHVLAYNMRADPSI-HDSTLDDQKSNDFVKTAS-------    51
                      *   :   : : :*.  :: *:.:

Sesbania              --------------------------DFMRT---------------VQIPENSKDE----    62
Lotus                 -------------------------QKLMRA---------------IHYLESRREQ----    65
Anthyllis             -------------------------DTYMTT---------------VHFPQVFHNLMART    68
Calophaca             ------------------------------SRR--------------TTNIPLDL-----    65
Astragalus            ------------------------------SRK--------------TTNMMI-------    61
Vicia                 KK---------------------D-MDYVKAPKI------------VSYEMPHEHPTY--    83
Pisum                 KK---------------------G-MDYVNAPKI------------VSYEMPLEDTTY--    83
Lathyrus              KK---------------------G-MDYVNAPKI------------VSYEMPLEHSTYGI    85
Cicer                 QNDNDSPTDETPAFQND---------QYSNDSPKTDAVSDNYHYTPYHYDFP-SE-----   102
Galega                ----GPPDEHIPPIIDGELDYQKGI-DYVKDKKID----VG-------------------    62
Trifolium             ----PPEPPTYPEINGEALDDQKVDQEYVKAQAKEEALDLNEQ-KVVEYNAQKIE-----    89
Trigonella            VDHNCPPGPTYPELSDSIIDDQKSI-DYVKDASTIDH-----------------K-----    90
Medicago_truncatula   -----------------------------------IA-----------------E-----    54
Medicago_sativa       -----------------------------------IA-----------------E-----    54

Sesbania              ------------------------------------------------------RIFHGI    68
Lotus                 -----------------------------------------------------GRKVTLM    72
Anthyllis             -------------------------------MNEEER---------------YKRDFNLM    82
Calophaca             --------------------------------SSDVALA-NR----------TQNSINAT    82
Astragalus            ------------------------------------------------------------    61
Vicia                 ------------------------------------------------------------    83
Pisum                 ------------------------------------------------------------    83
Lathyrus              FNEEKKKIGFEMIPTFENNTRPRRKKLAYLCLHELERLASKRTKDSYEDSTRWLTSTNSL   145
Cicer                 -----RPPLFNNI----------QESNDFPTTDETAA---------------FKNDQHAN   132
Galega                ------EQDY-------------QNGLDYVKAQKIDG---------------EQDYQKGL    88
Trifolium             -----EALGE-------------QKALEYVTAQKLEE---------------ILDDQKSQ   116
Trigonella            -----CPPGP-------------ST---Y--PELSDA---------------TLHDQKSI   112
Medicago_truncatula   -----CPPGP-------------HT---Y--PELSDS---------------TLNDQKSI    76
Medicago_sativa       -----CPPGP-------------DT---Y--LELSDS---------------TLDDQKSI    76

Sesbania              RRGSGTIL----------------------------------------------------    76
Lotus                 VPP---------------------------------------------------------    75
Anthyllis             DKDMNGLI----------------------------------------------------    90
Calophaca             EFE--------------------------------------------------ESRRTT-    91
Astragalus            ------------------------------------------------------------    61
Vicia                 ------------------------------------------------------------    83
Pisum                 ------------------------------------------------------------    83
Lathyrus              RYLKDKIS-RLSLKE------------LLTCV---------------------NSGEELN   171
Cicer                 DSPTTDAISDFYHDTPYHYEFPSDRTPYFNNIQDSNDSPKTDETPAFKNDQYSNDSPTTD   192
Galega                DYVKAQKI---------------------------------------------DG-----    98
Trifolium             EYVKTHKS---------------------------------------------EE-----   126
Trigonella            DYMKDASI---------------------------------------------DT-----   122
Medicago_truncatula   DYVKDA------------------------------------------------------    82
Medicago_sativa       DYVKDA------------------------------------------------------    82

Sesbania              ---------------------------EAATVKKH-------------------------    84
Lotus                 ------------------------------------------------------------    75
Anthyllis             ---------------------------SADELKAGMPKIAGKELTDEEVEQIISVLDLDG   123
Calophaca             -----------------NMPNGLS-------NDVAL---------DDLTEHFIKATELEE   118
Astragalus            -------------------PHGL-------------------------------------    65
Vicia                 --------------VAYEMPLEHTSSDI----------------NDEMIEEGRNDVDYVK   113
Pisum                 --------------VAYEMPLEHTSSSSAEINDGGLDYIK-VPRTDEVIEEHSKDVDYVK   128
Lathyrus              NKKGVDHKKAPK-FVAYEMPLEHTS--SSEINDGGLDYIK-VPRTDEVIEEHSK--DYVK   225
Cicer                 AVPDVDHNNP---YYYYEFPFEVA-----PPLFSNIQETNDSPTTDEAP-DFQKIQYSTD   243
Galega                --EQ-------------------------D-YEKGLDYVK-AQKIDEEQ-DYQKGLDYVK   128
Trifolium             --AL-------------------------D-EEMVLEYLKAAHKFAEAL-DDHKGQEYVK   157
Trigonella            --NTVDHNCPPGPSTYFKLNAWVQ-----D-DQNSINKVKD-PKTDEAM-RGQMGLEYEE   172
Medicago_truncatula   --STVDHNCPPGPHTHPELSVWLQ-----E-EQNSIDKVK-VLITDEAL-DNQKGLEYKD   132
Medicago_sativa       --STVDHNCPPGPHTYPELSAWLQ-----E-EQNSIDKVKKVLRTDEAP-DNQKGLEYKD   133
```

```
Sesbania                 -----------------------------FSYVPPA------------------------    91
Lotus                    AR--------------------TVHQQDY-------------------------------    84
Anthyllis                DR--------------------QISYEEFVKFLKTAKN---------------TRNVDD-    147
Calophaca                SRRTVN--IPHDLSSDC-KNDKVFR-QNPCETISGD----------ATGFAEKNDKI---    161
Astragalus               ------------------------------------------------------------    65
Vicia                    TPKNIAYEMPLEHTSYSDTNDEKFDDQKVLDFVKSPKTAAYEMPPESPTYSEINANLDEV    173
Pisum                    APKNVAYEMPLEHTSYSDINDEKFDNQKVLDFVKSPKTAAYEMPPESPTYPEINANLDEA    188
Lathyrus                 APKNVDYEMPLEHTSYSDINDEKFDDQKVLDFVKSPKTAAYEMPPESPTYPEINANLDEA    285
Cicer                    TSTIDS----------------TLN---------NQIIFDYTDPPVQ----------DD-    267
Galega                   AQKIDGIISEA--TSGI-------------------------------------------    143
Trifolium                AHKFAACESPPNPLTGLKINEEALDDQKDQEYVKAQKIAACESPPNPLTDHKINEALDD-    216
Trigonella               TPHKDG---PTEISTGTE----------------KENIPS--------------------    193
Medicago_truncatula      TRHNDG---SIEISTRTE----------------KDNFIPSDGPIEISSRTEKKNVIPS-    172
Medicago_sativa          ARHNDG---PIEISTGTE----------------KENFIPSDEPIEISTGTEKENFISS-    173

Sesbania                 ---------------------------------------------------EAEE-----    95
Lotus                    --------------------------------------------------VEGEG--EEF    92
Anthyllis                -------------------------------------------------QVKASE--RN-    155
Calophaca                -------------------------------LSEPK------------------------    166
Astragalus               -------------------------------SGEPK------------------------    70
Vicia                    LNDQKSVGSIKAQENDEKLDDQ-----------------------KSMDSMKAPQIDEKF    210
Pisum                    LNDQKGEDSLSAQKIDEKFDDNQKSIDSVESVNVPKIDEKFDDNQNGVDSVKASKIDEKL    248
Lathyrus                 LKDQKGEDSLNAQKMDKKFDDH-KSVDSVDLIKAPKIDEKLDDNQKNVDFV---------    335
Cicer                    -------------------------------------------DGPI---IEDETVTTKK    281
Galega                   ---------------------------------------------------------EKN    146
Trifolium                -------------------------------------------QKD----LEINGKAPK-    228
Trigonella               ------------------------------------------------------------    193
Medicago_truncatula      -------------------------------------------DES----IEISTGTEKE    185
Medicago_sativa          -------------------------------------------DEP----SEISSGAEKE    186

Sesbania                 RND-------------RNVKRSSS----------DEVDWILKP---KTIPPGSGTRRLLG    129
Lotus                    DVI-------------PNVKPSGVDW--KAFP-AAAYPWLKKDGAKTKTVPGNGNRRLLE    136
Anthyllis                VDD-------------HDVKPSGGDDRRTIFPAAAVYPWLKNDATKTKTT-IPGNRRLLE    201
Calophaca                -YYKDIY--------HMPPEGPEIDWRT----LIRLNPWRNINGGKT----IPGSRRLLK    209
Astragalus               -YYKDIS--IDNLIRTTMPPEPETFWMA----LYHANN-SKKNGAKT----IPGTRRLLR    118
Vicia                    DDQKGIDSMETPKSDETLNGQKGVEWIS----FHAKYPWIKKNS--F----TTGSRKLLL    260
Pisum                    DDEKDVDSSKFSKIDETLNDQKGVDWVS----FHARYPWIKKNS--L----TTGSRKLLL    298
Lathyrus                 ------DSVNAPKIDETLNDQKDVDWIS----FHAKYPWIKKNS--L----TTGSRKLLL    379
Cicer                    NDITT-------SESEKGEANKEANWVY----LHAKYPWMKKNVPKS----TTGTRKLLN    326
Galega                   NYD-------------TISSDSEAYWKT----FYAKYPWMKKNAPKT----IAGSRKLLL    185
Trifolium                -IAAASESSKPLTG--VEINESKVDWRS----FHAKYPWIKKNAHKS----IPGSRKLLN    277
Trigonella               --------------------EPQVDWRT----FHAKYPWIKKNDPNT----VTGSRKLLN    225
Medicago_truncatula      NFIPSVGPIDLTTGAQKKNFIPSVDWRT----FHAKYPWIKKNDPNT----VTGSRKLLN    237
Medicago_sativa          NFIPSVS---------------QVDWRT----FHAKYPWIKKDGPNT----VTGSRKLLN    223
                                                                              *.*:**

Sesbania                 D-FRHHIIKIPHHI--N  143
Lotus                    K-FGEEEIR--VLQLSN  150
Anthyllis                N-FGSEDDEMMVLQLSN  217
Calophaca                -DSGEEI----------  215
Astragalus               -EIKSP-----------  123
Vicia                    NHNANGQSA--------  269
Pisum                    NHIANDQSA--------  307
Lathyrus                 NHIANDESA--------  388
Cicer                    -DVANN*----------  331
Galega                   NDIEPMIYNDASRD---  199
Trifolium                -DIANDQLGGHSLQI--  291
Trigonella               -DIGEELN---------  232
Medicago_truncatula      -DIASE-----------  242
Medicago_sativa          -DIANK-----------  228
```

b

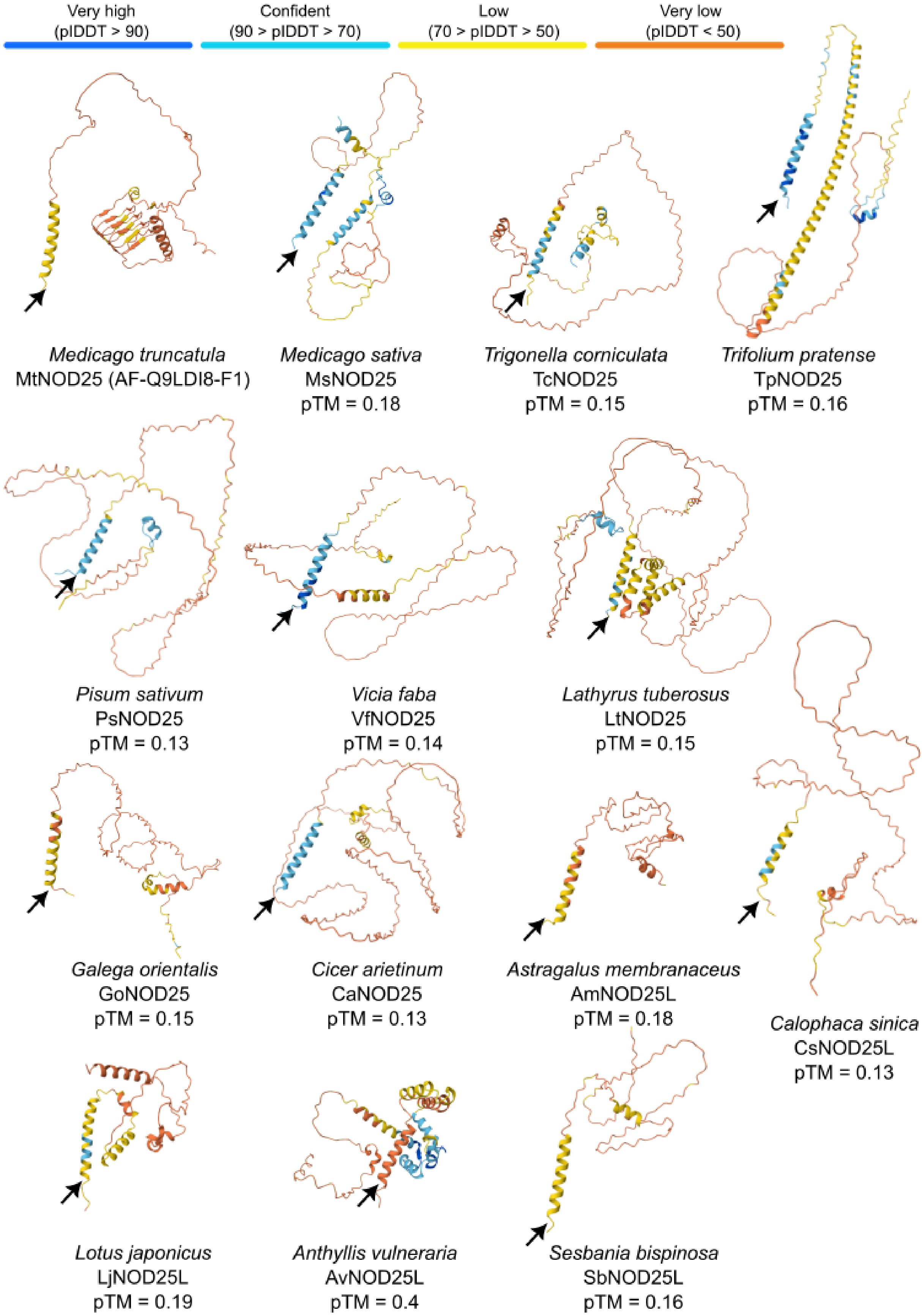

Fig. S1. (a) Multiple sequence alignment and structural predictions of NOD25 homologs. Sequences, identified from various legume species using the N- and C-terminal regions of *Medicago truncatula* NOD25 as queries, were aligned using CLUSTAL Omega (1.2.4). Conserved N terminal and C-terminal sequences are highlighted with the same colors as in Fig. 1. (b) Structural predictions of NOD25 homologs generated using AlphaFold3. The models exhibited low predicted template modeling (pTM) scores (see main text), indicating low confidence in the predicted structures. Arrows indicate the predicted signal peptides.

NAME	START	SITES	END	STRAND	MARGINAL SCORE
Medicago_tru	1	MVYSNSY.MFLGLGVFVLLSSHVLA	24	+	77.6
Trigonella	1	MVYSNSC.MFLGLGVFVLLSSHVLA	24	+	76.8
Medicago_sat	1	MVYSNSY.MFLGLGVFVLLSSHVLA	24	+	77.6
Trifolium	1	MVYSNSY.MFLGIGVFVILSSHALA	24	+	75.1
Pisum	1	MAYSNSY.ILFLLGVSVILSSQVLA	24	+	70.9
Lathyrus	1	MAYSNSY.MLFLLGVSLILSSHVLA	24	+	71.5
Vicia	1	MAYSNSY.MLFLLGVSLIISSQVLA	24	+	65.5
Galega	1	MAYSKPH.MLLCLGFFVILSSNVLV	24	+	51.7
Cicer	1	MDNSKSY.MLLCIGLFVILCSQVLA	24	+	59.2
Lotus	1	MASSKSYGLYLGLGLFVILFSQVFA	25	+	65.1
Anthyllis	1	MGSSKSCDLYLGLGLLAILFSQVFS	25	+	46.1
Sesbania	1	MAYSKSY.LCLGLGLFVILSSQALA	24	+	69.6
Calophaca	1	MAYSKSC.MPLVLGLFVILSSQVLA	24	+	67.6
Astragalus	1	MANLKTC.ISLGLGLLVILFSQVIA	24	+	43.5

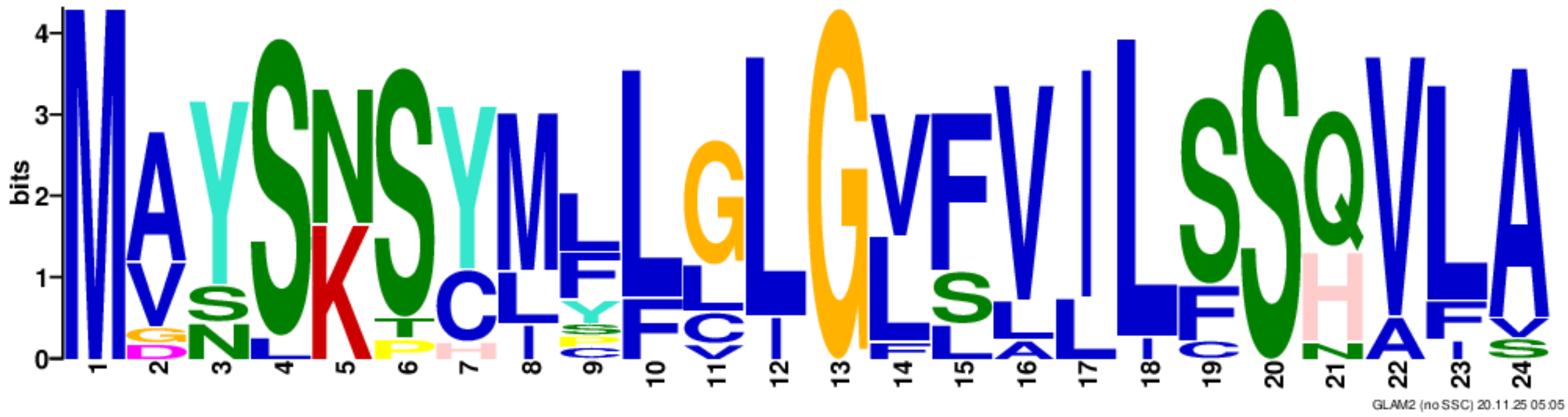

NAME	START	SITES	END	STRAND	MARGINAL SCORE
Medicago_tru	1	VDWRT.FHAK..YPWIKKNDPNTV....TGSRKLL.NDIASE	34	+	106.
Trigonella	1	VDWRT.FHAK..YPWIKKNDPNTV....TGSRKLL.NDIGEE	34	+	106.
Medicago_sat	1	VDWRT.FHAK..YPWIKKDGPNTV....TGSRKLL.NDIANK	34	+	104.
Trifolium	1	VDWRS.FHAK..YPWIKKNAHKSI....PGSRKLL.NDIAND	34	+	103.
Pisum	1	VDWVS.FHAR..YPWIKKNSL.T.....TGSRKLLLNHIAND	33	+	94.5
Lathyrus	1	VDWIS.FHAK..YPWIKKNSL.T.....TGSRKLLLNHIAND	33	+	96.4
Vicia	1	VEWIS.FHAK..YPWIKKNSF.T.....TGSRKLLLNHNANG	33	+	79.0
Galega	1	AYWKT.FYAK..YPWMKKNAPKTI....AGSRKLLLNDI.EP	34	+	75.8
Cicer	1	ANWVY.LHAK..YPWMKKNVPKST....TGTRKLL.NDVANN	34	+	69.5
Lotus	1	VDWKA.FPAAA.YPWLKKDGAKTKTVPGNGNRRLL.EKFGEE	39	+	60.3
Anthyllis	12	DDRRTIFPAAAVYPWLKNDATKTKTTI.PGNRRLL.ENFGSE	51	+	42.5
Calophaca	1	IDWRT.LI.RL.NPWRNINGGKTI....PGSRRLL.KDSGEE	34	+	30.8
Astragalus	1	TFWMALYHAN..NS..KKNGAKTI....PGTRRLL.REIKSP	33	+	23.2

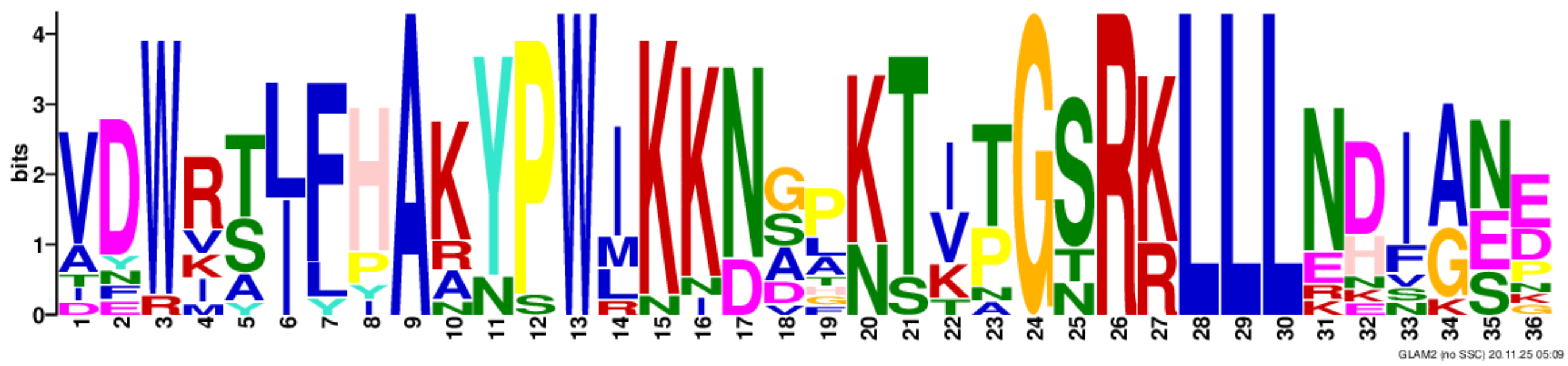

Figure S2. The alignment of conserved N- and C-terminal domains of NOD25-like sequences identified in legumes. N- and C-terminal sequences of NOD25-like sequences were aligned and the graphical representations of the sequence conservation of amino acids were generated using the method of Gapped Local Alignment of Motifs (GLAM2) specialized for short protein (Frith et al. 2008). The CLUSTAL color scheme was used for the alignments.

```
Medicago_sativa
  LENGTH = 170  COMBINED P-VALUE = 2.76e-44  E-VALUE =  3.9e-43
  DIAGRAM: [6]-2-[1]-[2]-2-[1]-2-[2]-2-[1]-4-[1]-1-[3]-3-[3]-3-[3]-5

     [6]          [1]            [2]               [1]              [2]
     1.6e-15      7.2e-13        1.4e-13           1.2e-13          1.0e-16
     YNMRADPSIHD  LDBQKGJDYVKAPKIYEMPPEPPTYPEJND  LDBQKGJDYVKAPKI  YEMPPEPPTYPEJ
     +++++++++++  ++++++ +++++++++++++++ +++++++  +++++++++++ +++  +++++++++++++
1    YNMRADPSIHDSTLDDQKSNDFVKTASIAECPPGPDTYLELSDSTLDDQKSIDYVKDASTVDHNCPPGPHTYPEL

         [1]                  [1]             [3]               [3]
         1.8e-05              1.6e-09         2.9e-19           3.9e-18
     ND  LDBQKGJDYVKAPKI      LDBQKGJDYVKAPKI GPIEISTGTEKENFI   GPIEISTGTEKENFI
     ++  + ++++++ ++            ++++++ +  ++++ +++++++++++++++   +++++++++++++++
76   SAWLQEEQNSIDKVKKVLRTDEAPDNQKGLEYKDARHNDGPIEISTGTEKENFIPSDEPIEISTGTEKENFISSD

     [3]
     5.1e-15
     GPIEISTGTEKENFI
     ++ ++++++++++++
151  EPSEISSGAEKENFIPSVSQ


Medicago_truncatula
  LENGTH = 184  COMBINED P-VALUE = 1.46e-40  E-VALUE =    2e-39
  DIAGRAM: [6]-2-[1]-[2]-2-[1]-2-[2]-2-[1]-3-[1]-1-[3]-3-[3]-3-[3]-3-[3]-2

     [6]          [1]            [2]               [1]              [2]
     1.6e-15      3.2e-12        4.5e-16           1.4e-12          3.3e-14
     YNMRADPSIHD  LDBQKGJDYVKAPKIYEMPPEPPTYPEJND  LDBQKGJDYVKAPKI  YEMPPEPPTYPEJ
     +++++++++++  ++++++ +++++++++++++++++++++++  + +++++++++ +++  +++++++++++++
1    YNMRADPSIHDSTLDDQMSNDFVKTASIAECPPGPHTYPELSDSTLNDQKSIDYVKDASTVDHNCPPGPHTHPEL

         [1]                [1]               [3]               [3]             [
         3.6e-08            1.6e-10           2.3e-16           6.1e-15         4
     ND  LDBQKGJDYVKAPKI    LDBQKGJDYVKAPKI   GPIEISTGTEKENFI   GPIEISTGTEKENFI G
     ++  + ++++++ +++   +   +++++++ +  ++++  +++++++++++++++   +++++++++++++ +   +
76   SVWLQEEQNSIDKVKVLITDEALDNQKGLEYKDTRHNDGSIEISTRTEKDNFIPSDGPIEISSRTEKKNVIPSDE

     3]                [3]
     .3e-17            7.2e-13
     PIEISTGTEKENFI    GPIEISTGTEKENFI
     ++++++++++++++    +++ +++++ +++++
151  SIEISTGTEKENFIPSVGPIDLTTGAQKKNFIPS


Trigonella corniculata
  LENGTH = 172  COMBINED P-VALUE = 6.34e-34  E-VALUE =  8.9e-33
  DIAGRAM: [6]-2-[1]-2-[2]-2-[1]-2-[2]-2-[1]-6-[2]-2-[1]-3-[1]-1-[3]-4

     [6]          [1]              [2]               [1]              [2]
     1.1e-13      6.1e-12          4.8e-10           5.6e-12          4.5e-15
     YNMRADPSIHD  LDBQKGJDYVKAPKI  YEMPPEPPTYPEJND  LDBQKGJDYVKAPKI  YEMPPGPPTYP
     +++++++++++  +++++++ +++++++    + ++ +++++++++    ++++++++++ +++  +++++++++++
1    YNMRADASIYDSTLDDQKSINYVNVASTTVDHNCPPGPTYPELSDSIIDDQKSIDYVKDASTIDHKCPPGPSTYP

           [1]                 [2]               [1]              [1]
           2.6e-11             9.8e-15           3.5e-10          1.8e-07
     EJND  LDBQKGJDYVKAPKI     YEMPPEPPTYPEJND   LDBQKGJDYVKAPKI  LDDQKGLDYVKAP
     ++++  + +++++++++ +++     +++++++++++++++   +++++++  ++ +++   + +++++ +  ++
76   ELSDATLHDQKSIDYMKDASIDTNTVDHNCPPGPSTYFKLNAWVQDDQNSINKVKDPKTDEAMRGQMGLEYEETP

        [3]
        3.3e-14
     KI GPIEISTGTEKENFI
     +  ++ +++++++++++
151  HKDGPTEISTGTEKENIPSEPQ


Cicer arietinum
  LENGTH = 273  COMBINED P-VALUE = 1.98e-28  E-VALUE =  2.8e-27
  DIAGRAM: 12-[1]-17-[4]-5-[8]-1-[4]-3-[4]-5-[8]-1-[4]-3-[4]-6-[8]-2-[4]-3-[4]-3-[1]-32

                 [1]                              [4]                [8]
                 2.7e-07                          7.3e-18            1.8e-19
                 LDBQKGJDYVKAPKI                  PAFKNIQYSNDSPTT    NYHDTPYHYEF
                 + +++++ + + + +                  +++++++++++++++    +++++++++++
1    YNDPVPPICDGELYYHKDLVYEKIPIIDGPPDVQNDNDSPTDETPAFQNDQYSNDSPKTDAVSDNYHYTPYHYDF

          [4]                  [4]                  [8]              [4]
          1.7e-16              1.9e-15              1.1e-20          4.4e-16
     PSER PAFKNIQYSNDSPTT      PAFKNIQYSNDSPTT      NYHDTPYHYEFPSER  PAFKNIQYSNDSPTT
     ++++ +++++++++++++++      +++++++++++++++      +++++++++++++++ +++++++ +++++++
76   PSERPPLFNNIQESNDFPTTDETAAFKNDQHANDSPTTDAISDFYHDTPYHYEFPSDRTPYFNNIQDSNDSPKTD
```

```
       [4]                  [8]              [4]               [4]
       1.3e-18              6.6e-05          1.7e-16           2.8e-11
       PAFKNIQYSNDSPTT      NYHDTPYHYEFPSER  PAFKNIQYSNDSPTT   PAFKNIQYSNDSPTT
       +++++++++++++++       +    + ++++ +   +++++++++++++++   + ++ ++++ ++ ++
151  ETPAFKNDQYSNDSPTTDAVPDVDHNNPYYYYEFPFEVAPPLFSNIQETNDSPTTDEAPDFQKIQYSTDTSTIDS

      [1]
      2.7e-06
      LDBQKGJDYVKAPKI
      + ++  +++   +
226  TLNNQIIFDYTDPPVQDDDGPIIEDETVTTKKNDITTSESEKGEANKE


Vicia faba
  LENGTH = 209  COMBINED P-VALUE = 3.92e-28  E-VALUE =  5.5e-27
  DIAGRAM: 1-[9]-2-[1]-3-[1]-2-[2]-14-[1]-2-[2]-2-[1]-2-[2]-5-[1]-3-[1]-3-[1]-9

      [9]          [1]               [1]              [2]
      3.1e-12      2.3e-16           7.4e-12          8.5e-13
      NRPSESSLNYG  LDBQKGJDYVKAPKI   LDBQKGJDYVKAPKI  YEMPPEPPTYPEJND
      +++++++++++  +++++++++++++++   + + +++++++++++  +++++++++++++++
1    YNRPSKSSLSYGEALDEQKGLDYVNAPKMYEELEAKKDMDYVKAPKIVSYEMPHEHPTYVAYEMPLEHTSSDIND

        [1]              [2]              [1]              [2]                 [
        7.5e-09          2.8e-12          1.2e-13          9.7e-16             2
        LDBQKGJDYVKAPKI  YEMPPEPPTYPEJND  LDBQKGJDYVKAPKI  YEMPPEPPTYPEJND     L
          + +++++++++++  ++++++++++++ ++  +++++++++++++++  +++++++++++++++     +
76   EMIEEGRNDVDYVKTPKNIAYEMPLEHTSYSDTNDEKFDDQKVLDFVKSPKTAAYEMPPESPTYSEINANLDEVL

     1]                [1]               [1]
     .9e-09            5.8e-13           6.7e-11
     DDQKGLDYVKAPKI    LDBQKGJDYVKAPKI   LDBQKGJDYVKAPKI
      +++++ +++++ +    +++++++++++++ +   ++++++++++ +++
151  NDQKSVGSIKAQENDEKLDDQKSMDSMKAPQIDEKFDDQKGIDSMETPKSDETLNGQKG


Pisum sativum
  LENGTH = 247  COMBINED P-VALUE = 5.18e-28  E-VALUE =  7.3e-27
  DIAGRAM: 1-[9]-2-[1]-3-[1]-2-[2]-10-[1]-4-[1]-2-[2]-2-[1]-2-[2]-5-[1]-4-[1]-7-[1]-3-[1]-9

      [9]          [1]               [1]              [2]                      [
      3.6e-13      2.3e-16           1.9e-12          1.7e-11                  2
      NRPSESSLNYG  LDBQKGJDYVKAPKI   LDBQKGJDYVKAPKI  YEMPPEPPTYPEJND          L
      +++++++++++  +++++++++++++++   + + +++++++++++  ++++++ ++++++++
1    YNRPSESSLNYGEELDEQKGLDYVNAPKMNEELEAKKGMDYVNAPKIVSYEMPLEDTTYVAYEMPLEHTSSSSAE

     1]                [1]              [2]              [1]              [2]
     .2e-08            1.8e-09          2.2e-13          1.3e-13          1.0e-1
     DDQKGLDYVKAPKI    LDBQKGJDYVKAPKI  YEMPPEPPTYPEJND  LDBQKGJDYVKAPKI  YEMPPE
      + +++++++++++        +++++++++++  +++++++++++++++  +++++++++++++++  ++++++
76   INDGGLDYIKVPRTDEVIEEHSKDVDYVKAPKNVAYEMPLEHTSYSDINDEKFDNQKVLDFVKSPKTAAYEMPPE

                   [1]                [1]                   [1]               [1
     6             2.0e-10            1.4e-08               8.9e-12           9.
     PPTYPEJND     LDBQKGJDYVKAPKI    LDBQKGJDYVKAPKI       LDBQKGJDYVKAPKI   LD
     +++++++++     + ++++ ++  ++++     +++++++++ +           +++++++++++ ++   ++
151  SPTYPEINANLDEALNDQKGEDSLSAQKIDEKFDDNQKSIDSVESVNVPKIDEKFDDNQNGVDSVKASKIDEKLD

     ]
     9e-09
     DQKGLDYVKAPKI
     + +++++ +  ++
226  DEKDVDSSKFSKIDETLNDQKG


Lathyrus tuberosus
  LENGTH = 328  COMBINED P-VALUE = 2.75e-26  E-VALUE =  3.9e-25
  DIAGRAM: 1-[9]-2-[1]-3-[1]-2-[2]-16-[9]-22-[9]-21-[1]-2-[2]-13-[1]-2-[2]-2-[1]-2-[2]-5-[1]-3-[1]-7-
[1]-12

      [9]          [1]               [1]              [2]
      8.7e-13      5.0e-16           1.9e-12          9.4e-13
      NRPSESSLNYG  LDBQKGJDYVKAPKI   LDBQKGJDYVKAPKI  YEMPPEPPTYPEJND
      +++++++++++  +++++++++++++++   + + +++++++++++  +++++++++++ +++
1    YNRPHESSLNYGEELDEQKGIDYVNAPKMNEELEAKKGMDYVNAPKIVSYEMPLEHSTYGIFNEEKKKIGFEMIP

          [9]                              [9]                             [1]
          1.6e-08                          3.4e-08                         4.1e-
          NRPSESSLNYG                      NRPSESSLNYG                     LDDQK
           +++++ ++++                      + +++++++++                     + + +
76   TFENNTRPRRKKLAYLCLHELERLASKRTKDSYEDSTRWLTSTNSLRYLKDKISRLSLKELLTCVNSGEELNNKK
```

```
                  [2]                         [1]              [2]              [
      10          1.3e-11                     2.0e-08          2.2e-13          1
      GLDYVKAPKI  YEMPPEPPTYPEJND             LDBQKGJDYVKAPKI  YEMPPEPPTYPEJND  L
      +++  +++++  +++++++++ +++++               +  + ++++++++  +++++++++++++++  +
151   GVDHKKAPKFVAYEMPLEHTSSSEINDGGLDYIKVPRTDEVIEEHSKDYVKAPKNVDYEMPLEHTSYSDINDEKF

      1]              [2]                 [1]               [1]
      .2e-13          1.0e-16             3.5e-10           7.5e-09
      DDQKGLDYVKAPKI  YEMPPEPPTYPEJND     LDBQKGJDYVKAPKI   LDBQKGJDYVKAPKI
      ++++++++++++++  +++++++++++++++     + ++++ ++ +++++   ++++++++++   +
226   DDQKVLDFVKSPKTAAYEMPPESPTYPEINANLDEALKDQKGEDSLNAQKMDKKFDDHKSVDSVDLIKAPKIDEK

       [1]
       3.1e-08
       LDBQKGJDYVKAPKI
        ++++ ++++ +
301   LDDNQKNVDFVDSVNAPKIDETLNDQKD


Calophaca sinica
  LENGTH = 156  COMBINED P-VALUE = 1.81e-21  E-VALUE =  2.5e-20
  DIAGRAM: 12-[5]-8-[5]-8-[5]-56

                  [5]                             [5]
                  4.3e-30                         3.0e-30
                  VALANRTQNSINATEFEESRRTTN        VALANRTQNSINATEFEESRRTTN
                  ++++++++++++++++++++++++        ++++++++++++++++++++++++
1     HKVPETLVPISDVALANHTQNSINATEFEESRRTTNIPLDLSSDVALANRTQNSINATEFEESRRTTNMPNGLSN

       [5]
       2.5e-20
       VALANRTQNSINATEFEESRRTTN
       ++++++++++++++++++++++++
76    DVALDDLTEHFIKATELEESRRTVNIPHDLSSDCKNDKVFRQNPCETISGDATGFAEKNDKILSEPKYYKDIYHM


Trifolium pratense
  LENGTH = 224  COMBINED P-VALUE = 1.51e-15  E-VALUE =  2.1e-14
  DIAGRAM: 12-[2]-3-[1]-22-[1]-3-[1]-3-[1]-4-[1]-2-[2]-2-[1]-2-[2]-36

                  [2]               [1]                                  [1]
                  1.5e-15           7.3e-05                              1.9e-09
                  YEMPPEPPTYPEJND   LDBQKGJDYVKAPKI                      LDDQKGLD
                  +++++++++++++++    +   ++ +++++                        + +++ +
1     NHLASEPLIYDVYKMPPEPPTYPEINGEALDDQKVDQEYVKAQAKEEALDLNEQKVVEYNAQKIEEALGEQKALE

                [1]               [1]                [1]              [2]
                8.9e-12           8.6e-09            1.9e-12          4.9e-11
      YVKAPKI   LDBQKGJDYVKAPKI   LDBQKGJDYVKAPKI    LDBQKGJDYVKAPKI  YEMPPEPPTYP
      ++ +++    +++++++ ++++++    +++ +++ + ++++     +++++++ +++++++  +++++++++++
76    YVTAQKLEEILDDQKSQEYVKTHKSEEALDEEMVLEYLKAAHKFAEALDDHKGQEYVKAHKFAACESPPNPLTGL

            [1]              [2]
            1.1e-13          1.2e-10
      EJND  LDBQKGJDYVKAPKI  YEMPPEPPTYPEJND
      ++++  +++++++ +++++++  +++++++++ +++++
151   KINEEALDDQKDQEYVKAQKIAACESPPNPLTDHKINEALDDQKDLEINGKAPKIAAASESSKPLTGVEINESK


Astragalus membranacus
  LENGTH = 66  COMBINED P-VALUE = 4.75e-12  E-VALUE =  6.7e-11
  DIAGRAM: 10-[5]-32

                [5]
                1.0e-18
                VALANRTQNSINATEFEESRRTTN
                ++++++++++++++++++++ +++
1     DKSPEAICDSSLLSCGSKNSVEVAEFEESRKTTNMMIPHGLSGEPKYYKDISIDNLIRTTMPPEPE


Galega orientalis
  LENGTH = 132  COMBINED P-VALUE = 3.53e-09  E-VALUE =  4.9e-08
  DIAGRAM: 20-[1]-4-[1]-3-[1]-3-[1]-3-[1]-24

                          [1]                [1]               [1]
                          8.4e-14            4.1e-15           2.0e-16
                          LDBQKGJDYVKAPKI    LDBQKGJDYVKAPKI   LDBQKGJDYVKAPKI
                          +++++++++++  ++    +++++++++++++++   +++++++++++++++
1     DKVNCVGPPDEHIPPIIDGELDYQKGIDYVKDKKIDVGEQDYQNGLDYVKAQKIDGEQDYQKGLDYVKAQKIDGE

      [1]               [1]
      6.7e-14           2.0e-16
      LDBQKGJDYVKAPKI   LDBQKGJDYVKAPKI
      +++ +++++++++++   +++++++++++++++
76    QDYEKGLDYVKAQKIDEEQDYQKGLDYVKAQKIDGIISEATSGIEKNNYDTISSDSE
```

```
Sesbania bispinosa
  LENGTH = 73  COMBINED P-VALUE = 2.81e-07  E-VALUE =  3.9e-06
  DIAGRAM: 12-[7]-51

                 [7]
                 4.8e-13
                 GRKMVLDGRM
                 ++++++++++
1    LQLPDAEATLPPGRKMTLDGRMEDFMRTVQIPENSKDERIFHGIRRGSGTILEAATVKKHFSYVPPAEAEERN


Anthyllis vulneraria
  LENGTH = 130  COMBINED P-VALUE = 7.38e-06  E-VALUE =   0.0001
  DIAGRAM: 11-[7]-109

                [7]
                5.2e-12
                GRKMVLDGRM
                ++++++++++
1    QTLPQNEVNNLGEKMVMDGRMSVDTYMTTVHFPQVFHNLMARTMNEEERYKRDFNLMDKDMNGLISADELKAGMP


Lotus japonicus
  LENGTH = 77  COMBINED P-VALUE = 1.55e-04  E-VALUE =   0.0022
  DIAGRAM: 12-[7]-55

                 [7]
                 2.9e-10
                 GRKMVLDGRM
                  +++++++++
1    HTLTEADKAADPERKMVLDRRINMQKLMRAIHYLESRREQGRKVTLMVPPARTVHQQDYVEGEGEEFDVIPNVKP
```

Figure S3. Repeat modules identified in NOD25-like proteins in different legume species. Highlighted sequences represent distinct repeat modules identified by MEME analysis of the internal domains of MtNOD25-like proteins and correspond to the schematic representation and color scheme shown in Fig. 1. The Repeat 1, Repeat 2 and Repeat 3 modules, identified in a previous study (Hohnjec et al. 2009) correspond to repeats [2], [1] and [3], respectively in this study and are highlighted on *M. truncatula* sequences with brown, light yellow and light green.

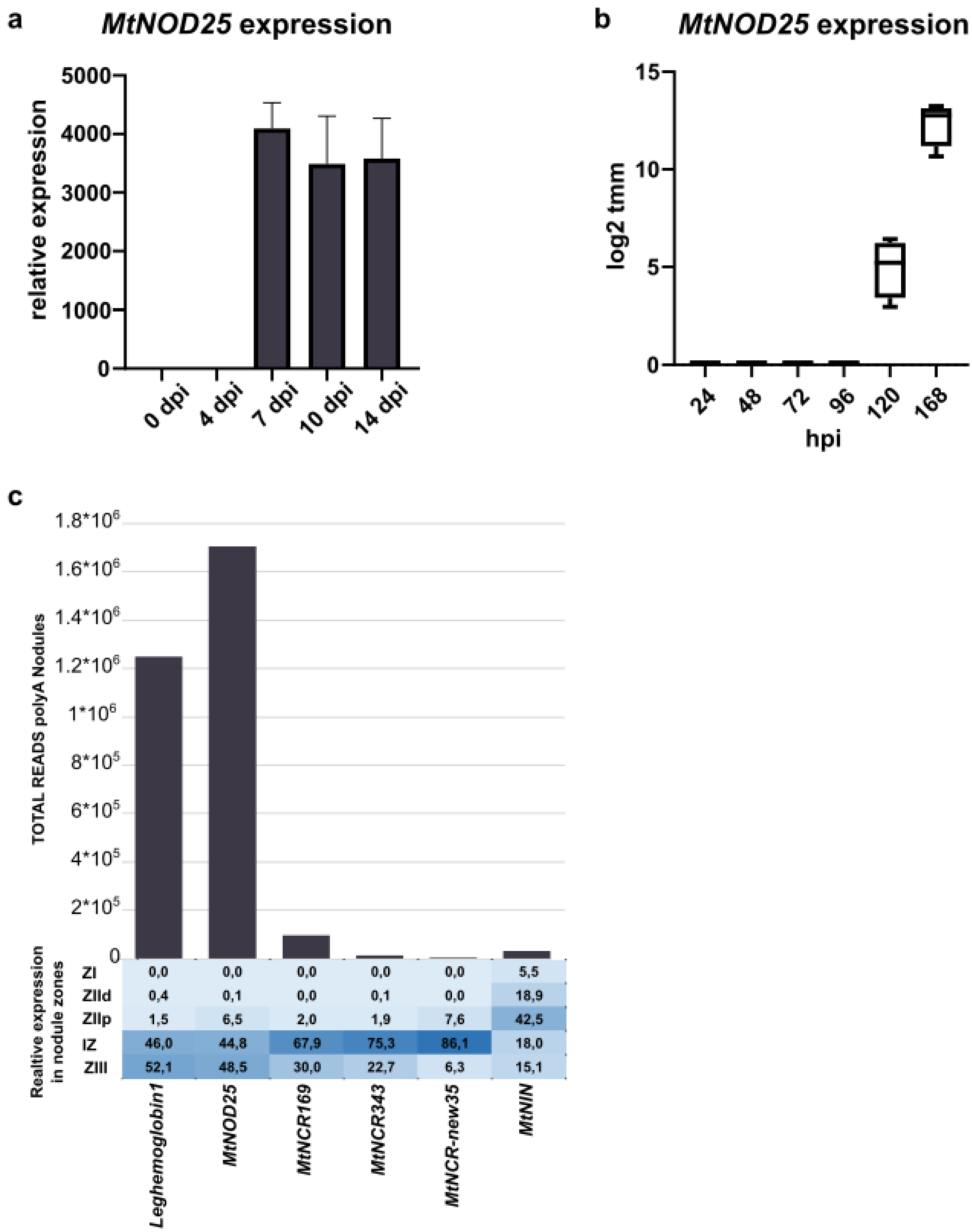

Figure S4. Transcriptional activity of *MtNOD25* in wild-type *M. truncatula* nodules. a) Expression analysis of *MtNOD25* gene during nodule development. The relative expression of *MtNOD25* was analyzed by reverse transcription quantitative polymerase chain reaction (RT-qPCR) in wild-type (WT) nodules at 0, 4, 7, 10, and 14 days post-inoculation (dpi) with *S. medicae* strain WSM419 m2011. (**b**) The temporal activity of *MtNOD25* expression was also monitored in the early stage of rhizobia interaction (24-168 hours post inoculation) based on Affymetrix GeneChip data in the Medicago truncatula Gene Expression Atlas (Benedito et al. 2008). *MtNOD25* is induced 5 days post inoculation

with *S. meliloti* 1021. Error bars indicate SE. (**c**) Relative spatial expression of *Medicago truncatula Leghemoglobin1*, *NOD25*, *NCR169, NCR343, NCR-new35* and *NIN* genes generated with RNA sequencing of different nodule zones obtained with laser-capture microdissection described in the study by Roux et al. (2014). *Leghemoglobin1* (Medtr5g066070 - genome release v. Mt4.0; MtrunA17_Chr5g0427351 - MtrunA17r5.0) and *NOD25* are extremely highly expressed in the interzone and nitrogen fixation zone of *M. truncatula* nodules compared with *NCR* peptide genes and the transcription regulator *NIN* (MtrunA17_Chr5g0448621). ZI: zone I (meristem); ZIId: invasion zone distal; ZIIp: invasion zone proximal; IZ: interzone; ZIII nitrogen-fixation zone.

TAAGTTTAGCCACACAAATATTTTAGGAACATGAGAATTTATACTTGATAACTTCGTCTAGTCACTTCTTTCCAAGAAGGAC

TCTTTATTATTCAAGCATAAGATACAAATTTGTGTCTTTTAAAGAACTAATTGTCATCACTTGACATATATACAATTATTACA

AGAAAAAATATTTGCTTCATACGATATCAATGTTG **.. Δ1304 bp..**GTAACATTGTTTTGCAGTTAACTCTTCTTAAATCCATG

GTTGTTTTTTACGATCTTAATTAACTAGTCCATAAATTTTAAAAATCAAATCATAGATATTTCAACTAACTTTTTTTTTTTTTTG

ACAAAATAAAAGATATTTATTCATTCAAATTGATAGAGTACATCGATATAATGCAAATTCGCTAAAAACAAAAAGGA

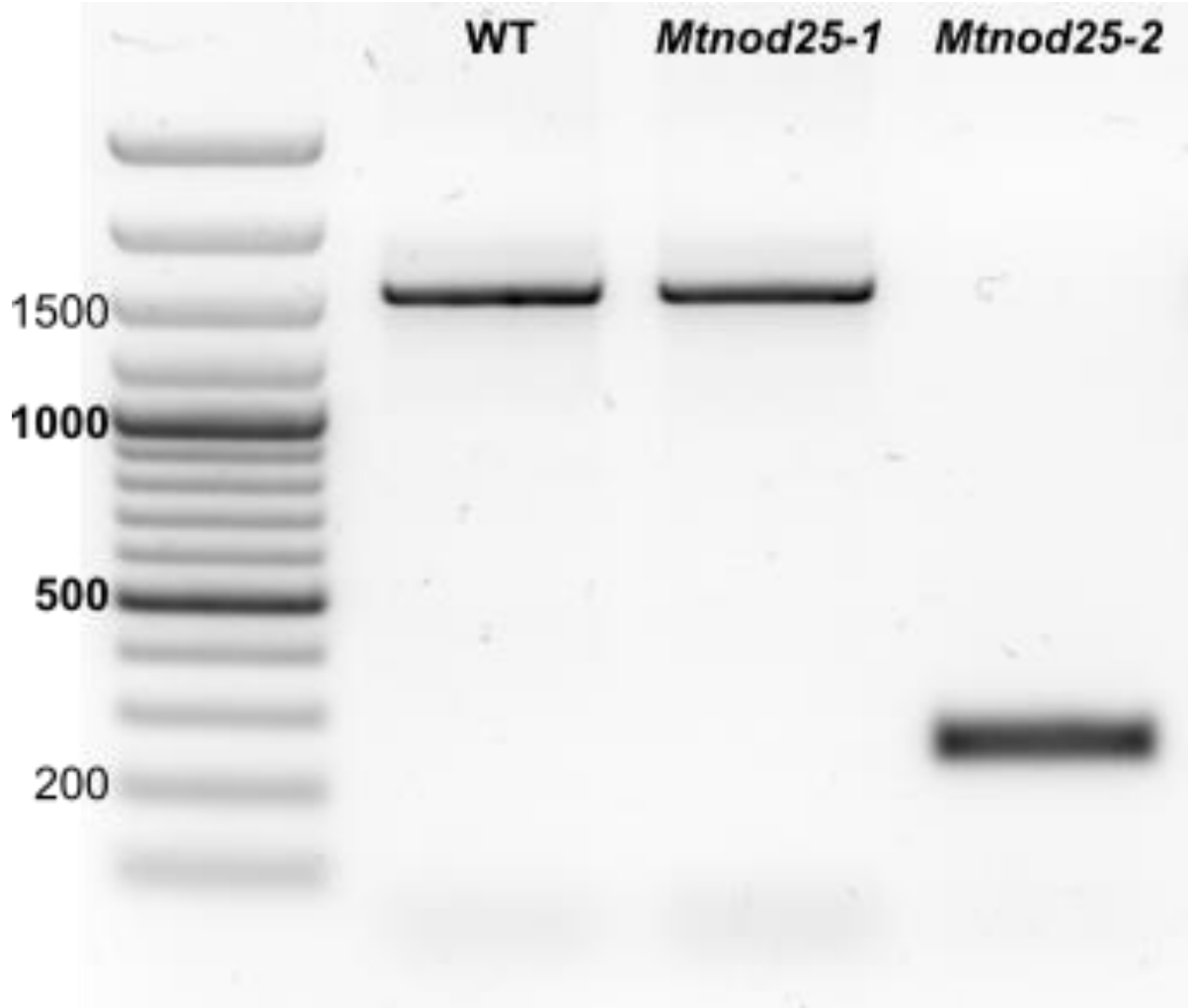

Figure S5. Position of the deletion in the *Mtnod25-2* mutant. PCR products amplified using primer pair Nod25_F10 (shaded in turquoise) and Nod25_R10 (yellow) were sequenced to identify the position and the size of the deletion in *Mtnod25-2* allele. The deletion extends from the promoter region to the second intron of *MtNOD25*. In wild-type and *Mtnod25-1* mutant plants, the amplified DNA fragments span 1,568 bp, whereas in *Mtnod25-2* the PCR product is reduced to 264 bp.

a

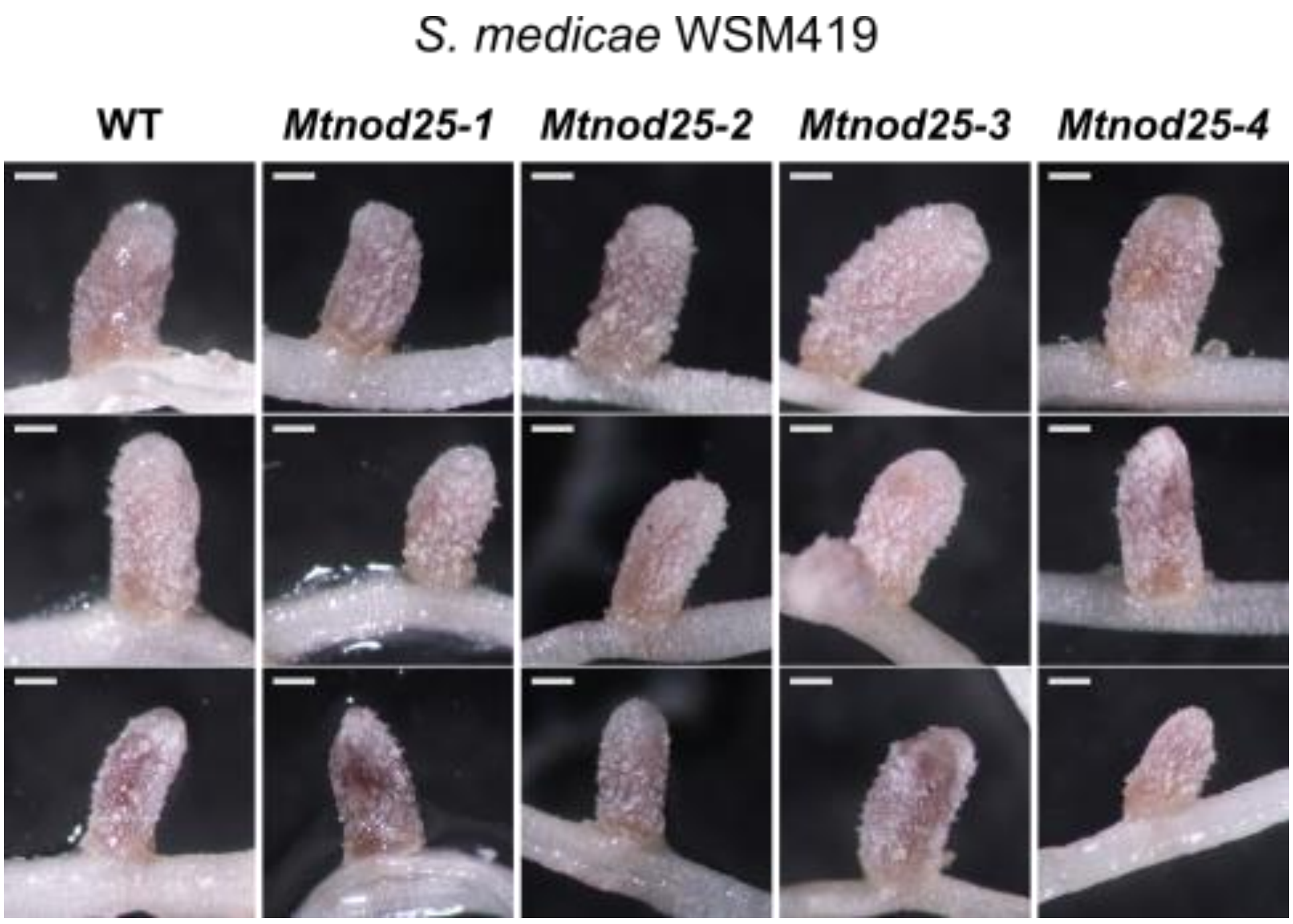

b

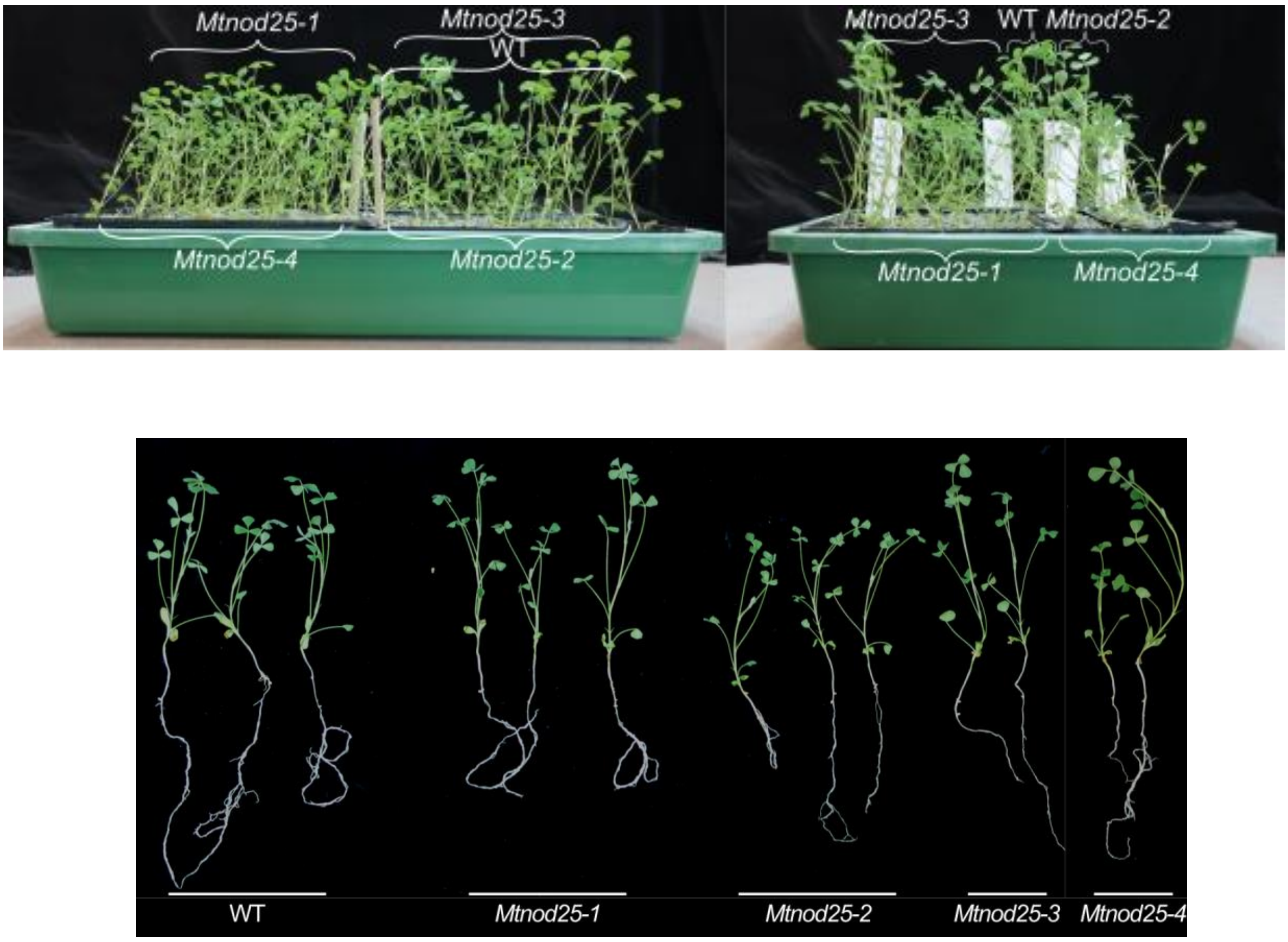

Figure S6. Nodulation phenotype and growth of the *Mtnod25* mutant lines. (a) Three weeks post inoculation with *S. medicae* WSM419, both wild-type and mutant plants developed elongated and pink nodules characteristic of functional symbiosis. Scale bar: 0.5 mm (b) The overall growth of the four *Mtnod25* mutant alleles was comparable with wild-type plants.

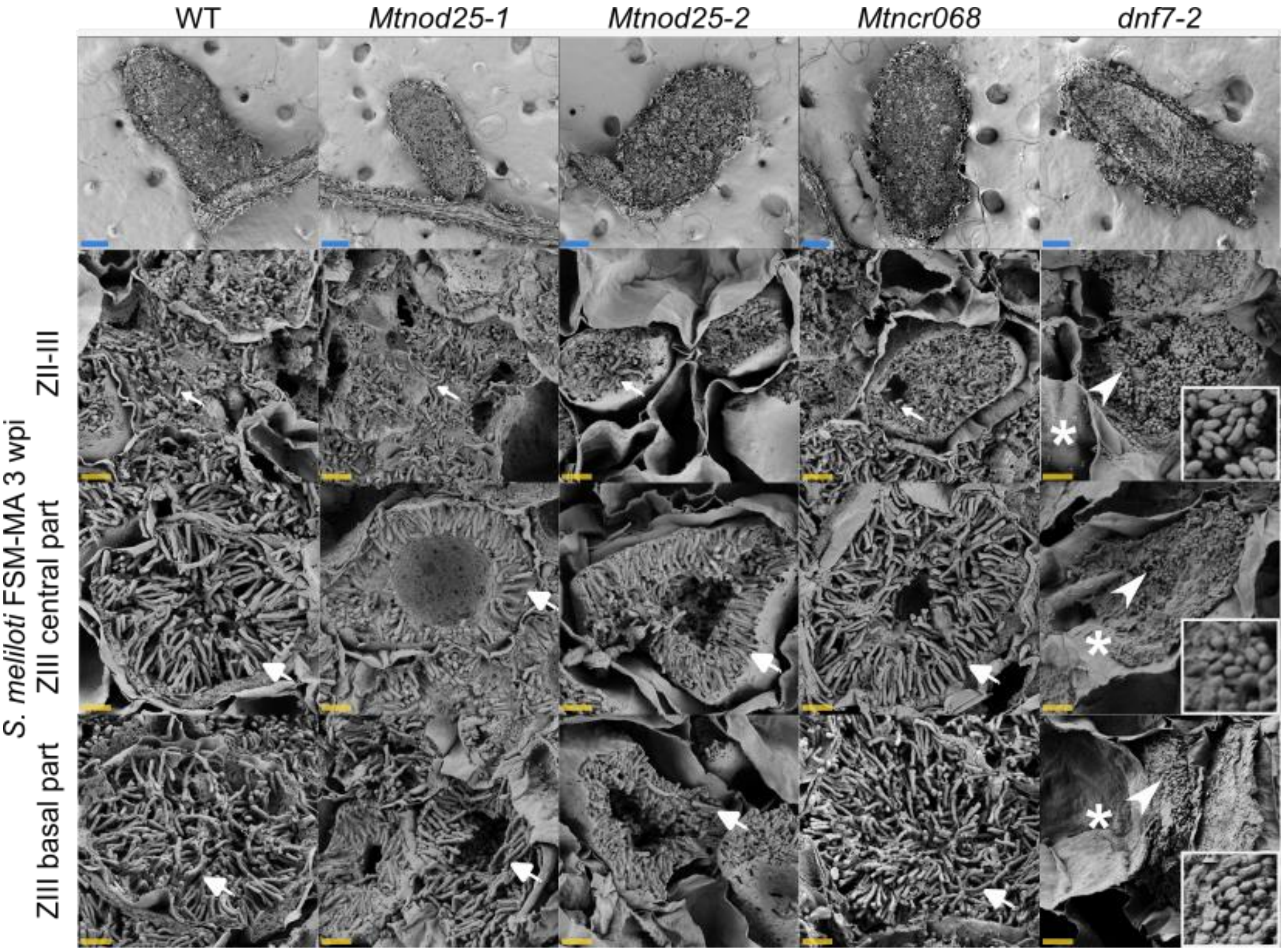

Figure S7. **Symbiotic cells of *Mtnod25* nodules are indistinguishable from wild-type nodule cells.** Scanning electron micrographs of nodules from wild-type, *Mnod25-1*, *Mtnod25-2*, *Mtncr068* and *dnf7-2* plants harvested 3 weeks post inoculation with *S. meliloti* strain FSM-MA. Bacterial morphology was assessed in high-magnification images of cells from the interzone (ZII-III) and the central or basal regions of nitrogen fixation zones (ZIII). Undifferentiated, round-shaped rhizobia were present in both ZII and ZIII, consistent with the defective symbiosis phenotype *dnf7-2* nodules. In contrast, nodules of wild-type, *Mtncr068* and *Mtnod25* mutants contained differentiated bacteroids, positioned around the central vacuoles, Undifferentiated, round-shaped rhizobia (arrowheads) were observed in ZII-III and ZIII of the symbiosis defective *dnf7-2* nodules, whereas differentiated bacteroids (small and large arrows), positioned around the central vacuoles, were present in nodules of wild-type, *Mtncr068* and *Mtnod25* mutant plants. Asterisks show empty symbiotic cells in *dnf7-2*. Blue scale bars: 200 µm, yellow bars: 5 µm
